# tRF3a-MetCAT Promotes EGFR-Targeted Therapeutic Resistance through the TRIM21–STAT1–C5a Axis in Lung Adenocarcinoma

**DOI:** 10.34133/research.0911

**Published:** 2025-10-07

**Authors:** Yuchen Zhang, Pingjing Zhou, Yifan Guo, Hongyu Zhang, Jie Gu, Di Ge, Guangyin Zhao

**Affiliations:** ^1^ Department of Thoracic Surgery, Zhongshan Hospital, Fudan University, Shanghai 200032, China.; ^2^ Department of Thoracic Surgery, Shanghai Geriatric Medical Center, Shanghai 201104, China.

## Abstract

Despite the effectiveness of epidermal growth factor receptor tyrosine kinase inhibitors (EGFR-TKIs) in treating lung adenocarcinoma with epidermal growth factor receptor (EGFR) mutations, many patients eventually stop responding to therapy. This resistance typically arises when bypass signaling pathways become activated, undermining the treatment’s efficacy. The function of transfer-RNA-derived small RNAs, a novel class of regulatory noncoding RNAs, is poorly understood in EGFR-TKI resistance. This study demonstrates that osimertinib-resistant lung cancer organoid models with EGFR mutations (19del or L858R) exhibit marked up-regulation of tRF3a-MetCAT, which correlates with poor prognosis. tRF3a-MetCAT promotes lung cancer cell proliferation, migration, and invasion in vitro while conferring osimertinib resistance. Mechanistically, tRF3a-MetCAT attenuates the interaction between the E3 ubiquitin ligase tripartite motif-containing 21 (TRIM21) and signal transducer and activator of transcription 1 (STAT1), resulting in STAT1 stabilization and transcriptional up-regulation of the downstream target C5. Elevated C5a levels subsequently activate the extracellular signal-regulated kinase signaling pathway, contributing to drug resistance. In vivo, treatment with the C5a-targeting inhibitor eculizumab or the C5a receptor inhibitor PMX53 effectively mitigates tRF3a-MetCAT-induced osimertinib resistance. These results reveal novel resistance pathways in EGFR-mutant lung cancer and suggest therapeutic approaches to addressing EGFR-TKI resistance.

## Introduction

Lung adenocarcinoma (LUAD) is the most common histological subtype of lung cancer, and it frequently harbors mutations in the epidermal growth factor receptor (EGFR) gene, which are among the most prevalent genetic alterations in this malignancy [1]. Upon activation, EGFR, a membrane-associated receptor tyrosine kinase, initiates downstream tyrosine phosphorylation cascades that promote tumor cell proliferation. By specifically targeting EGFR activation, EGFR tyrosine kinase inhibitors (TKIs) interrupt this signaling cascade [2]. The results of the AURA3 trial indicate that osimertinib offers considerable efficacy and safety in clinical practice [3]. Nevertheless, owing to tumor heterogeneity and intrinsic complexity, a considerable proportion of patients exhibit primary resistance or eventually acquire resistance to osimertinib [4,5]. These resistance mechanisms often include the activation of alternative signaling routes like the extracellular signal-regulated kinase (ERK) signaling pathway [6].

Once dismissed as mere cellular debris from transfer RNA (tRNA) breakdown, transfer-RNA-derived small RNAs (tsRNAs) have emerged as key players in gene regulation. These molecules, generated from precursor or mature tRNAs under stressful conditions, are now recognized as biologically functional regulators rather than accidental byproducts [7–9]. tsRNAs exhibit differential expression profiles in various tumors and modulate multiple oncogenic processes, including cell growth, invasion, metastasis, immune modulation, therapy insensitivity, and metabolic transformation, through interactions with both RNAs and proteins [10–12]. Despite growing evidence supporting the involvement of tsRNAs in tumor progression, their functional contribution to resistance to EGFR-targeted therapies remains largely unexplored. We hypothesized that specific tsRNAs play critical roles in mediating EGFR-TKI resistance and that targeting these tsRNAs could introduce an innovative treatment approach to combating drug resistance in EGFR-mutant LUAD.

The complement system is essential for innate immunity. Upon activation, complement proteins acquire enzymatic activity and mediate immune responses and inflammation, thereby playing vital roles in host defense [13,14]. However, dysregulated complement activation may trigger immune dysfunction and autoimmune disorders [15]. C5a, a powerful pro-inflammatory peptide, is one of the main elements of the complement cascade generated by C5 convertase cleavage of C5 [16]. C5a is secreted extracellularly and functions by interacting with its receptor, C5aR (C5a receptor) [17]. Within the tumor immune microenvironment, multiple immune cell types, such as neutrophils and natural killer cells, can be modulated by complement activation, leading to immunosuppressive effects that promote tumor progression and evasion [18–20]. In addition to its immune regulatory functions, the complement system can act directly on cancer. The complement system’s role in promoting tumor growth has been observed in colorectal cancer and melanoma, suggesting the potential therapeutic utility of focusing on this biological route [21–23]. However, the interplay between the complement system and targeted therapy in EGFR-mutant lung cancers remains largely unexplored.

In this study, we established osimertinib-resistant LUAD organoid models and performed tsRNA sequencing to identify resistance-associated tsRNAs. We identified tRF3a-MetCAT as a candidate tsRNA strongly linked to EGFR therapy resistance. Functional assays in both vitro and vivo conditions showed that tRF3a-MetCAT stimulates EGFR-mutated LUAD cell growth, mobility, and infiltration while providing resistance to osimertinib. Mechanistically, tRF3a-MetCAT binds to tripartite motif-containing 21 (TRIM21) and interferes with its interaction with signal transducer and activator of transcription 1 (STAT1), thereby reducing STAT1 ubiquitination and enhancing its stability. Stabilized STAT1 acts as a transcriptional activator of C5, leading to increased C5 transcription and subsequent activation of the C5a/C5aR/ERK signaling pathway. This bypass of downstream EGFR signaling ultimately results in resistance to osimertinib. Based on these findings, we propose that focusing on C5a may serve as a promising therapeutic strategy for LUAD patients who exhibit high tRF3a-MetCAT expression and resistance.

## Results

### tRF3a-MetCAT is highly expressed in EGFR-TKI-resistant LUAD

**Fig. 1.**
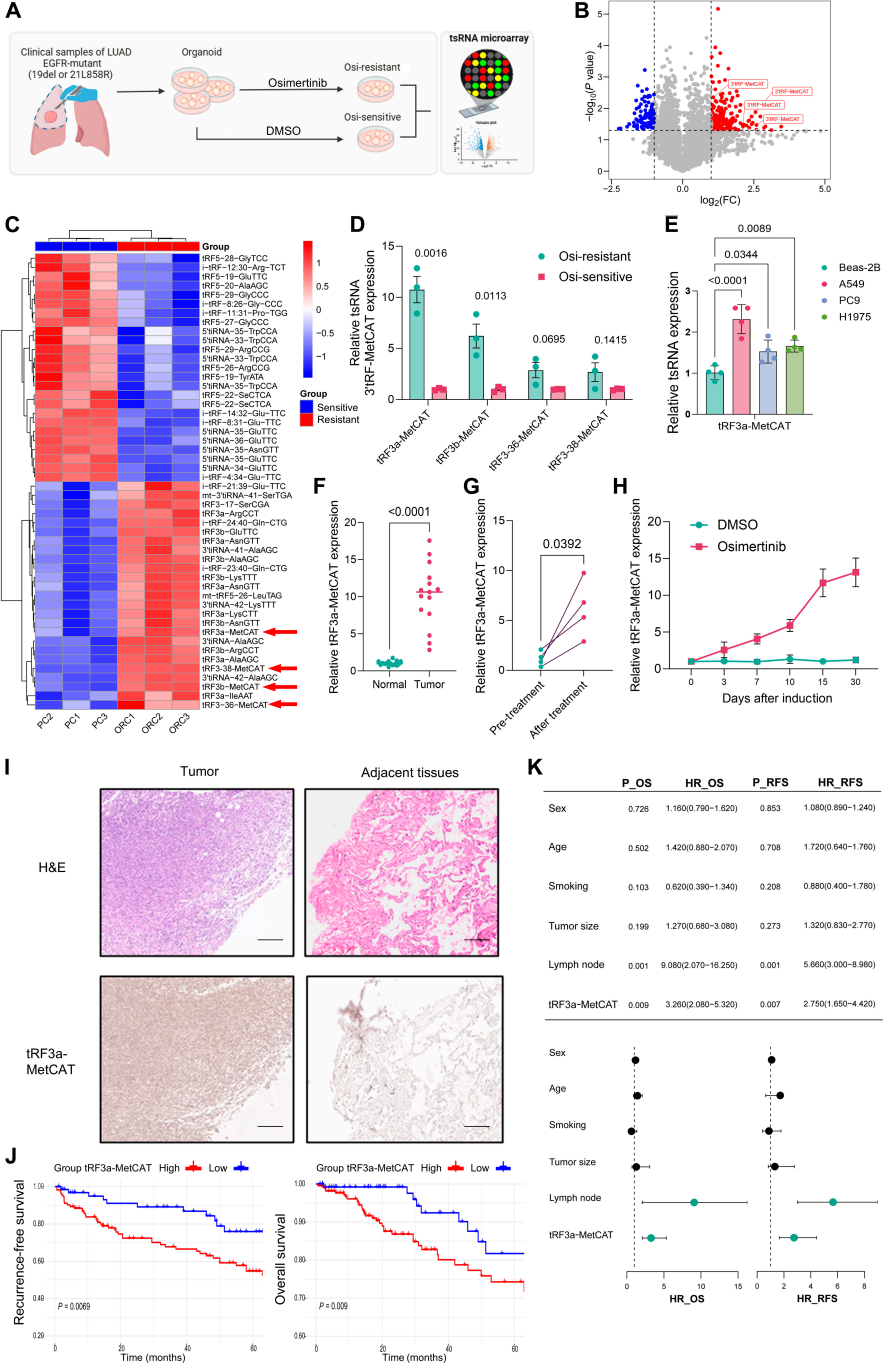
tRF3a-MetCAT overexpression in osimertinib-resistant lung adenocarcinoma (LUAD) tissues associated with poor prognosis. (A) Schematic diagram of the construction and sequencing workflow of osimertinib-resistant lung cancer organoids. (B) Volcano plot of transfer-RNA-derived small RNAs (tsRNA) microarray sequencing results. (C) Heatmap of differentially expressed tsRNAs based on sequencing data. (D) Reverse transcription quantitative polymerase chain reaction (RT-qPCR) validation of candidate tsRNA expression levels in organoids. (E) RT-qPCR analysis of tRF3a-MetCAT expression in normal bronchial epithelial cells (Beas-2B) and various lung cancer cell lines (A549, PC-9, and H1975). (F) RT-qPCR detection of tRF3a-MetCAT expression in LUAD tissues and paired adjacent normal lung tissues. (G) RT-qPCR analysis of tRF3a-MetCAT expression in paired LUAD tissues before and after osimertinib treatment. (H) Relative expression of tRF3a-MetCAT in LUAD organoids during treatment with osimertinib or dimethyl sulfoxide (DMSO), assessed by RT-qPCR (organoids derived from the same 3 patients shown in (A)). (I) In situ hybridization (ISH) detection of tRF3a-MetCAT expression in LUAD tissues and paired adjacent normal tissues, accompanied by corresponding hematoxylin and eosin (H&E) staining. Scale bar: 10 μm. (J) Kaplan–Meier analysis of recurrence-free survival (left) and overall survival (right) based on tRF3a-MetCAT expression levels in a validated LUAD cohort (*n* = 120). (K) Forest plot showing hazard ratios and 95% confidence intervals from univariable logistic regression analysis in a cohort of 120 LUAD cases. Osi, osimertinib; FC, fold change; P, *P* value; HR, hazard ratio; OS, overall survival; RFS, recurrence-free survival.

To explore the function of tsRNAs in the context of response to precision medicine in EGFR-mutated LUAD, we collected surgical tumor specimens from patients with EGFR exon 19 deletions or L858R point mutations in exon 21 and established patient-derived organoid models. Organoids were treated with either osimertinib or vehicle control (dimethyl sulfoxide) to generate resistant and sensitive groups, respectively, followed by tsRNA microarray sequencing (Fig. 1A). The sequencing results revealed significant up-regulation of a class of tsRNAs derived from methionine-tRNA, the tRF-MetCAT family, in the resistant group (Fig. 1B and C). Among 4 enriched tRF-MetCAT subtypes, tRF3a-MetCAT exhibited the most pronounced differential expression between the resistant and sensitive organoids (Fig. 1D).

### tRF3a-MetCAT expression is increased in LUAD specimens and associated with poor clinical outcomes

tRF3a-MetCAT levels were notably reduced in normal lung epithelial cell lines compared to those in the cancer cell lines (Fig. 1E). Similarly, tumor tissues exhibited higher levels of tRF3a-MetCAT in fresh clinical samples than they did in adjacent nontumor tissues, as determined by quantitative polymerase chain reaction (qPCR) (Fig. 1F). We further classified these samples into pre-WT, pre-Mut, and Res-Mut groups. tRF3a-MetCAT levels were higher in tumors than in adjacent tissues across all groups and significantly elevated in Res-Mut tumors than in both pre-WT and pre-Mut tumors (Fig. S1A). In paired tumor samples, tRF3a-MetCAT levels were lower in pre-treatment biopsies than in those collected after osimertinib treatment (Fig. 1G). Moreover, during organoid model resistance development, tRF3a-MetCAT expression progressively increased with prolonged exposure to osimertinib, suggesting its drug-induced up-regulation (Fig. 1H). In situ hybridization (ISH) confirmed higher expression of tRF3a-MetCAT in tumor regions than in adjacent tissues (Fig. 1I). Analysis of a tissue microarray comprising 120 LUAD specimens further revealed that high tRF3a-MetCAT levels correlated strongly with unfavorable outcomes (Fig. 1J and K). These findings suggest that tRF3a-MetCAT not only is linked to targeted therapy resistance in EGFR-mutant LUAD but may also serve as a prognostic indicator of poor clinical outcomes.

### tRF3a-MetCAT overexpression promotes resistance to osimertinib in EGFR-mutant LUAD in vitro and in vivo

**Fig. 2.**
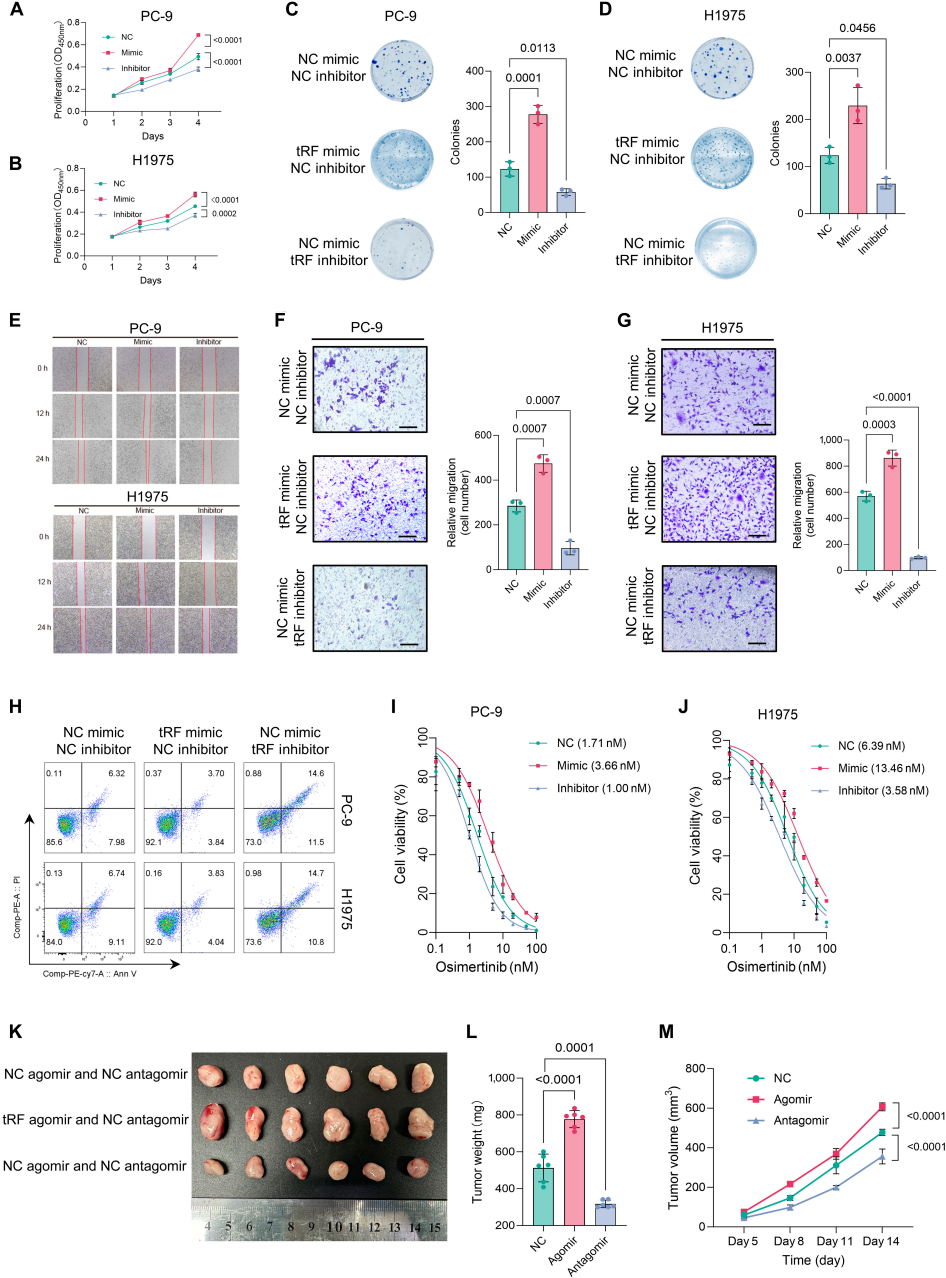
Overexpression of tRF3a-MetCAT promotes proliferation and metastasis and reduces epidermal growth factor receptor tyrosine kinase inhibitor (EGFR-TKI) sensitivity in LUAD cells. (A and B) Proliferation rates of PC-9 (A) and H1975 cells (B) overexpressing or inhibiting tRF3a-MetCAT measured at the indicated times. (C and D) Representative images and quantification of colony formation of PC-9 (C) and H1975 cells (D) with overexpression or inhibition of tRF3a-MetCAT. (E) Representative images of wound healing assays. (F and G) Representative images and quantification of invasion assays in PC-9 (F) and H1975 cells (G) with overexpression or inhibition of tRF3a-MetCAT. (H) Representative flow cytometry plots of apoptosis analysis in PC-9 and H1975 cells with overexpression or inhibition of tRF3a-MetCAT. (I and J) Relative cell viability of PC-9 (I) and H1975 cells’ (J) overexpression or inhibition of tRF3a-MetCAT treated with osimertinib for 5 d. (K to M) Representative images of tumor xenografts with PC-9 cells overexpressing or inhibiting tRF3a-MetCAT into a nude mouse (K). Tumor weight (L) and volume (M) were determined and analyzed (*N* = 5 mice per group). NC, negative control; tRF, tRF3a-MetCAT.

To explore tRF3a-MetCAT’s biological role, we established overexpression and knockdown models using 2 LUAD cell lines harboring EGFR mutations, PC-9 and H1975. tRF3a-MetCAT overexpression markedly boosted proliferation (Fig. 2A and B), colony growth (Fig. 2C and D), migration (Fig. 2E), and invasiveness (Fig. 2F and G) in both cell lines. Decreased apoptosis was observed in tRF3a-MetCAT-overexpressing cells (Fig. 2H). Subsequent treatment with osimertinib revealed that overexpression of tRF3a-MetCAT markedly reduced the sensitivity of PC-9 and H1975 cells to the drug, whereas knockdown of tRF3a-MetCAT restored sensitivity (Fig. 2I and J).

We assessed tRF3a-MetCAT’s impact on tumor progression in vivo via a xenograft mouse model. Tumors derived from PC-9 cells overexpressing tRF3a-MetCAT by agomir treatment exhibited significantly accelerated growth compared with control tumors (Fig. 2K to M). Collectively, these results indicate that tRF3a-MetCAT potentiates resistance to EGFR inhibitors in LUAD harboring EGFR mutations by enhancing proliferation, migration, and invasion while also facilitating tumor progression in vivo.

### tRF3a-MetCAT activates the complement pathway and promotes C5a expression

**Fig. 3.**
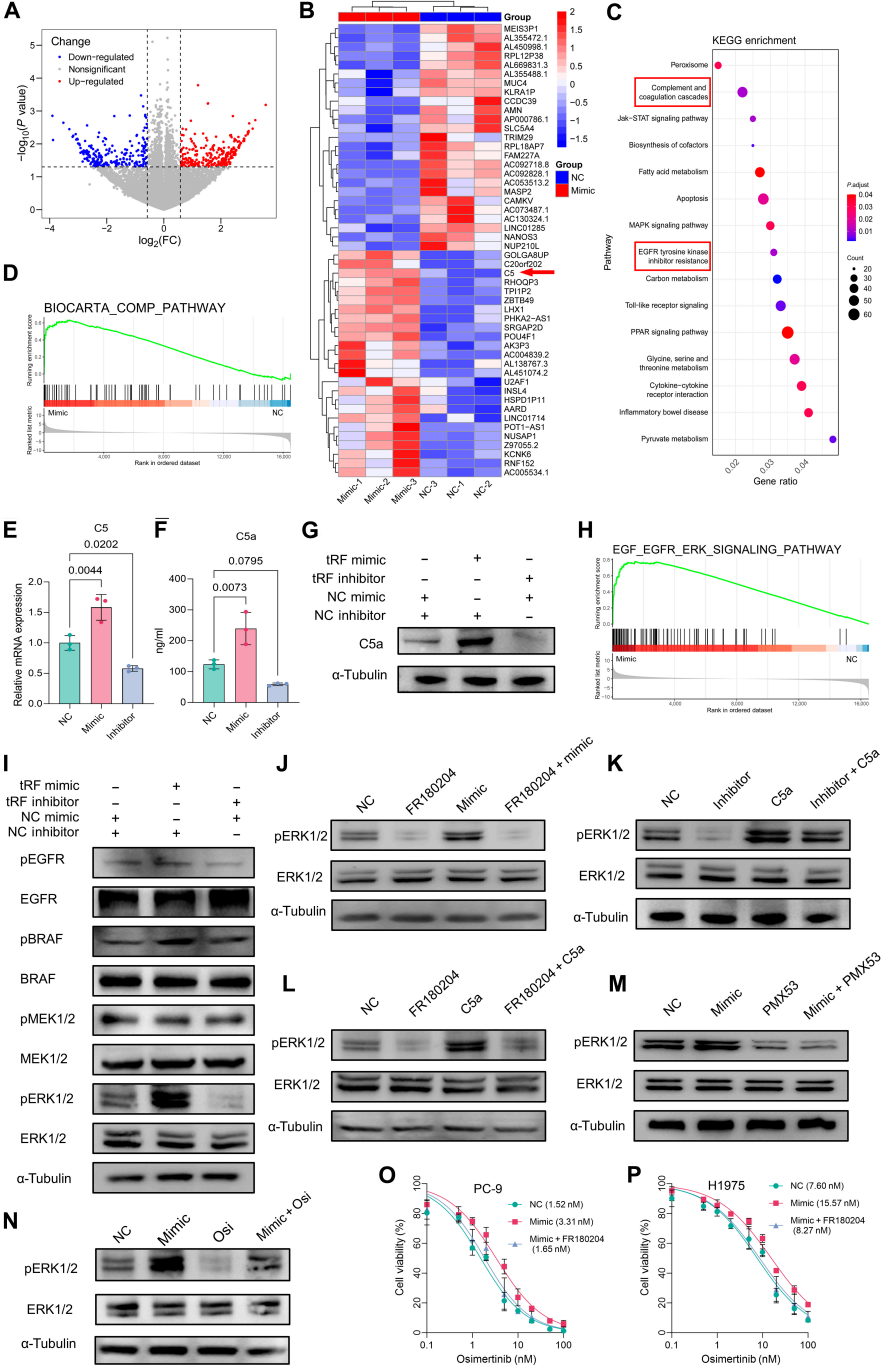
tRF3a-MetCAT induces C5 transcription and C5a secretion, triggering extracellular signal-regulated kinase (ERK) pathway activation. (A) Volcano plot of RNA sequencing (RNA-seq) results comparing PC-9 cells overexpressing tRF3a-MetCAT with control cells. (B) Heatmap of differentially expressed genes from RNA-seq analysis, with C5 indicated by an arrow. (C) Kyoto Encyclopedia of Genes and Genomes (KEGG) pathway analyses of up-regulated differential genes in PC-9 cells overexpressing tRF3a-MetCAT compared with control cells. (D) Gene set enrichment analysis (GSEA) showing significant enrichment of the complement pathway. (E) RT-qPCR analysis of C5 expression in PC-9 cells overexpressing or inhibiting tRF3a-MetCAT. (F) Enzyme-linked immunosorbent assay (ELISA) analysis of C5a levels in PC-9 and H1975 cells overexpressing or inhibiting tRF3a-MetCAT. (G) Western blot analysis of C5a levels in PC-9 cells overexpressing or inhibiting tRF3a-MetCAT. (H) GSEA enrichment of the EGFR–ERK signaling pathway in PC-9 cells overexpressing tRF3a-MetCAT. (I) Western blot analysis of the EGFR–BRAF–MEK–ERK signaling pathway in PC-9 cells overexpressing or inhibiting tRF3a-MetCAT. (J) Western blot analysis of ERK phosphorylation in PC-9 cells overexpressing tRF3a-MetCAT, with or without FR180204 treatment (30 μM for 48 h). (K) Western blot analysis of ERK phosphorylation in PC-9 cells with tRF3a-MetCAT inhibition, with or without recombinant C5a treatment (20 μg/ml for 24 h). (L) Western blot analysis of ERK phosphorylation in PC-9 cells treated with FR180204, recombinant C5a, or both. (M) Western blot analysis of ERK phosphorylation in PC-9 cells overexpressing tRF3a-MetCAT, with or without PMX53 treatment (2 μM for 24 h). (N) Western blot analysis of ERK phosphorylation in PC-9 cells overexpressing tRF3a-MetCAT, with or without osimertinib treatment (5 nM for 24 h). (O and P) Relative cell viability of indicated PC-9 (O) and H1975 cells (P) treated with osimertinib for 5 d. mRNA, messenger RNA. BRAF, B-Raf proto-oncogene, serine/threonine kinase; MEK, mitogen-activated protein kinase kinase.

To clarify how tRF3a-MetCAT drives resistance to osimertinib, we performed RNA sequencing on PC-9 cells overexpressing tRF3a-MetCAT and compared them with control cells. tRF3a-MetCAT overexpression up-regulated several genes, including C5, ZBTB49, and RNF152, whereas TRIM29 and SLC5A4 were down-regulated (Fig. 3A and B). Pathway enrichment study revealed considerable enrichment within the complement cascade, the peroxisome pathway, fatty acid metabolism, apoptosis, and EGFR-TKI resistance pathways (Fig. 3C). Gene set enrichment analysis (GSEA) subsequently validated the marked activation of the complement cascade in tRF3a-MetCAT-overexpressing cell lines (Fig. 3D).

The complement system is essential for innate immunity, with complement components C3 and C5 playing pivotal roles in its activation. We examined the transcriptional levels of C3 and C5 following tRF3a-MetCAT overexpression or knockdown and found notable changes, particularly in C5 (Fig. 3E and Fig. S2A). Upon transcription, C5 is cleaved into C5a and C5b; C5a is secreted into the extracellular space and exerts biological effects by binding to its receptor C5aR, whereas C5b contributes to membrane attack complex formation. We measured the C5a levels in both the culture supernatant and cell lysates and observed a marked increase in C5a expression following tRF3a-MetCAT overexpression (Fig. 3F and G). However, the expression of its receptor C5aR remained unchanged (Fig. S2B). Collectively, these results indicate that tRF3a-MetCAT activates the complement signaling pathway and promotes C5a but not C5aR expression, which may contribute to acquired resistance to osimertinib.

### tRF3a-MetCAT enhances LUAD resistance to EGFR-TKIs through ERK pathway activation mediated by C5a

GSEA indicated significant enrichment of the EGFR–ERK signaling pathway following tRF3a-MetCAT overexpression (Fig. 3H). Western blotting verified that tRF3a-MetCAT selectively increased ERK1/2 phosphorylation, indicating pathway activation (Fig. 3I). This effect was abolished by treatment with FR180204, a selective ERK1/2 phosphorylation inhibitor, indicating that tRF3a-MetCAT-induced ERK activation was phosphorylation dependent (Fig. 3J).

Given the previous reports that C5a/C5aR signaling can trigger downstream ERK1/2 phosphorylation [24], we examined the relationship between C5a and ERK activation in this context. Supplementation with recombinant C5a reversed the suppression of ERK1/2 phosphorylation caused by tRF3a-MetCAT knockdown, whereas FR180204 blocked C5a-induced ERK activation (Fig. 3K and L). Treatment with the C5aR inhibitor PMX53 suppressed the increase in ERK1/2 phosphorylation induced by tRF3a-MetCAT overexpression, indicating that C5aR is required for ERK activation in this condition (Fig. 3M). These results support a model in which C5a, through its receptor C5aR, acts as a key effector linking tRF3a-MetCAT to ERK pathway activation. As osimertinib suppressed tumor growth primarily through the inhibition of the EGFR–ERK axis, we observed that tRF3a-MetCAT overexpression partially reversed the suppressive effect of osimertinib on ERK1/2 phosphorylation (Fig. 3N). Importantly, the increased resistance to osimertinib observed in tRF3a-MetCAT-overexpressing EGFR-mutant LUAD cells was abrogated by ERK inhibition, highlighting the essential role of ERK1/2 phosphorylation in mediating drug resistance (Fig. 3O and P). Similarly, blockade of C5aR produced a comparable effect, further supporting the involvement of the C5a–C5aR–ERK axis in resistance (Fig. S2C and D). Together, these findings demonstrate that tRF3a-MetCAT promotes acquired resistance to EGFR-TKI therapy in LUAD by up-regulating C5a, which activates ERK1/2 phosphorylation and downstream signaling.

### tRF3a-MetCAT directly binds TRIM21 and regulates C5a transcriptional activation

**Fig. 4.**
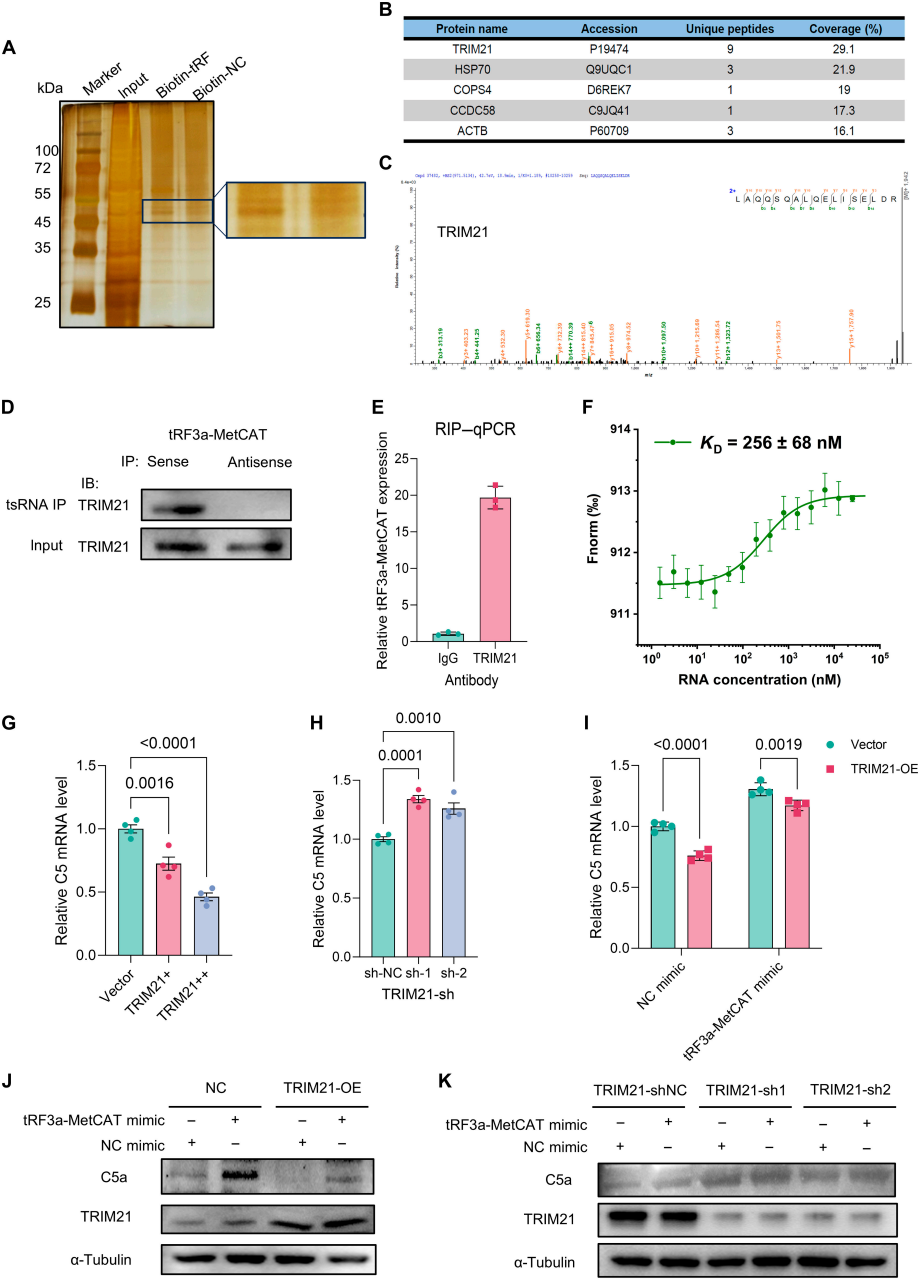
tRF3a-MetCAT directly binds to tripartite motif-containing 21 (TRIM21) and modulates C5/C5a expression. (A) Silver sodium dodecyl sulfate–polyacrylamide gel electrophoresis (SDS-PAGE) image revealing proteins immunoprecipitated by tRF3a-MetCAT and its antisense RNA in PC-9-OR cells. (B) Mass spectrometry analysis identifying potential tRF3a-MetCAT-interacting proteins. (C) TRIM21 was identified as a candidate binding partner in the mass spectrometry dataset. (D) Western blot analysis of proteins pulled down by tRF3a-MetCAT confirms the interaction with TRIM21. (E) RNA immunoprecipitation (RIP) followed by RT-qPCR verifies the binding of tRF3a-MetCAT to TRIM21. (F) Microscale thermophoresis (MST) confirmed direct binding between TRIM21 and tRF3a-MetCAT. (G and H) RT-qPCR analysis of C5 expression after TRIM21 overexpression (G) or knockdown (H) in PC-9 cells. (I) RT-qPCR analysis of C5a expression in control or tRF3a-MetCAT-overexpressing PC-9 cells following TRIM21 overexpression. (J and K) Western blot analysis of C5a protein levels in control or tRF3a-MetCAT-overexpressing PC-9 cells upon TRIM21 overexpression (J) or knockdown (K). IP, immunoprecipitation; IB, immunoblotting; IgG, immunoglobulin G; TRIM21-OE, TRIM21 overexpression; Fnorm, normalized fluorescence.

Typically, tsRNAs, like many long noncoding RNAs, mediate their regulatory functions via binding to RNAs or proteins [25,26]. Given that tRF3a-MetCAT up-regulates C5a expression, we investigated whether it directly binds to the messenger RNA (mRNA) of C5. However, sequence alignment and structural prediction revealed no potential base-pairing interactions between tRF3a-MetCAT and C5 mRNA. To identify the protein interactors of tRF3a-MetCAT, we performed RNA pull-down assays using a biotin-labeled tRF3a-MetCAT probe, followed by mass spectrometry (Fig. 4A). This approach revealed that TRIM21 was significantly enriched in the pull-down complex (Fig. 4B and C). Western blotting confirmed interaction between tRF3a-MetCAT and TRIM21 (Fig. 4D). Moreover, RNA immunoprecipitation (RIP) using TRIM21 antibody successfully pulled down endogenous tRF3a-MetCAT in PC-9 cells (Fig. 4E). To validate the direct interaction between TRIM21 and tRF3a-MetCAT, we performed microscale thermophoresis (MST) analysis, which confirmed specific binding with a dissociation constant of *K*_D_ = 256 ± 68 nM, supporting a direct physical association between the 2 molecules (Fig. 4F).

Next, we examined whether TRIM21 regulates C5a expression. As TRIM21 is a well-characterized E3 ubiquitin ligase, we initially hypothesized that it modulates C5a stability via ubiquitination. However, co-immunoprecipitation (Co-IP) and cycloheximide chase assays showed no interaction between TRIM21 and C5a excluding the possibility of posttranslational regulation (Fig. S3A to D).

TRIM21 overexpression decreased the transcriptional level of C5, whereas TRIM21 knockdown led to its increase (Fig. 4G and H). This effect was diminished in the presence of tRF3a-MetCAT, indicating that tRF3a-MetCAT interferes with TRIM21-mediated transcriptional regulation of C5 (Fig. 4I). Notably, tRF3a-MetCAT did not affect TRIM21 expression, suggesting that other factors are also involved in the process by which tRF3a-MetCAT regulates the transcriptional level of C5 through TRIM21 (Fig. S3E). At the protein level, the effects of TRIM21 overexpression on C5a expression were similarly attenuated by tRF3a-MetCAT and were nearly abolished when TRIM21 was knocked down (Fig. 4J and K). Collectively, these findings demonstrate that TRIM21 is a key regulatory mediator of C5 transcription and that tRF3a-MetCAT impairs this regulatory axis by directly binding to TRIM21, thereby promoting C5a expression and contributing to EGFR-TKI resistance.

### STAT1 functions as a transcription factor for C5 in EGFR-mutant LUAD cells

**Fig. 5.**
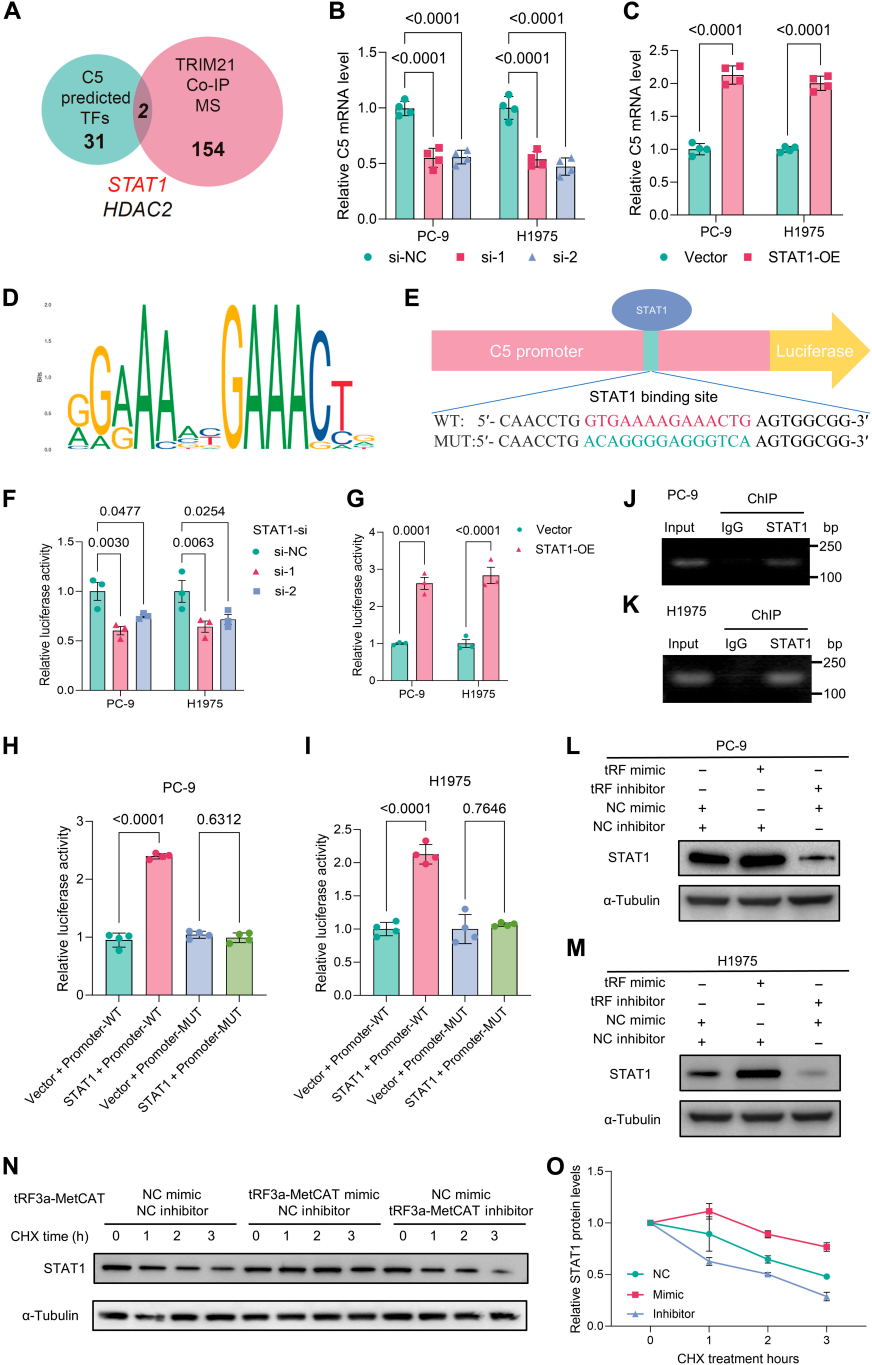
tRF3a-MetCAT enhances signal transducer and activator of transcription 1 (STAT1) protein stability to promote C5 transcription. (A) Venn diagram showing the intersection between the predicted transcription factors of C5 and TRIM21-interacting proteins identified by co-immunoprecipitation (Co-IP) and mass spectrometry. (B and C) RT-qPCR analysis of C5 mRNA levels following STAT1 knockdown (B) or overexpression (C) in PC-9 cells. (D and E) Schematic diagrams showing predicted STAT1-binding motifs within the C5 promoter region based on JASPAR database analysis. (F) Luciferase activity of the C5 promoter reporter in STAT1 knockdown (si-1 and si-2) or control (si-NC) PC-9 and H1975 cells. (G) Luciferase activity of the C5 promoter reporter in STAT1 overexpression (STAT1-OE) or control (Vector) PC-9 and H1975 cells. (H and I) Luciferase activity of the C5 promoter reporter (Promoter-WT) with the indicated STAT1 binding site mutated C5 promoter (Promoter-Mut) in STAT1-overexpressing (STAT1) or control (Vector) PC-9 (H) and H1975 cells (I). (J and K) Chromatin immunoprecipitation (ChIP)–PCR analysis of the interaction between STAT1 and the C5 promoter in PC-9 (J) and H1975 cells (K). (L and M) Western blot analysis of the STAT1 expression levels in PC-9 (L) and H1975 cells (M) overexpressing or inhibiting tRF3a-MetCAT. (N and O) Half-life analysis of STAT1 after overexpression or inhibition of tRF3a-MetCAT treated with cycloheximide (CHX; 40 μg/ml) for the indicated times in PC-9 cell lines (N). The quantifications are shown on the right (O). TFs, transcription factors; MS, mass spectrometry.

Given that both tRF3a-MetCAT and TRIM21 regulate C5 expression at the transcriptional level, we hypothesized that transcription factors are involved in this regulatory process. To identify potential candidates, we predicted the transcription factors of C5 and compared the results with proteins identified by TRIM21 Co-IP mass spectrometry analysis (Fig. 5A). Compared to STAT1, knockdown of HDAC2 did not significantly reduce the mRNA expression of C5 (Fig. 5B and Fig. S4A). In addition, analysis of publicly available chromatin immunoprecipitation (ChIP) sequencing datasets revealed potential STAT1 binding peaks at the promoter region of the C5 gene (Fig. S4D). Therefore, we selected STAT1 for further investigation (Fig. S4E). Functional validation showed that STAT1 overexpression significantly increased C5 transcription (Fig. 5C and Fig. S4C). Using JASPAR, we predicted that the promoter region of C5 contains a STAT1-binding motif (Fig. 5D and E) [27]. A dual-luciferase reporter assay confirmed that STAT1 activated C5 transcription, whereas mutation of the STAT1-binding motif abolished this activation (Fig. 5F to I). Furthermore, ChIP followed by PCR demonstrated STAT1’s direct interaction with the C5 gene’s promoter area (Fig. 5J and K). These findings collectively identified STAT1 as a transcription factor for C5 in EGFR-mutant LUAD cells.

### tRF3a-MetCAT regulates STAT1 stability

To investigate whether tRF3a-MetCAT modulates C5 transcription through STAT1, we examined STAT1 expression under conditions of tRF3a-MetCAT overexpression or inhibition. We found that tRF3a-MetCAT overexpression led to increased STAT1 protein levels, whereas its inhibition had the opposite effect (Fig. 5L and M). Notably, this change was observed only at the protein level with no significant alteration in STAT1 mRNA expression, suggesting a posttranscriptional regulatory mechanism (Fig. S5A). Further analysis revealed that tRF3a-MetCAT overexpression prolonged the half-life of STAT1, thereby enhancing its stability (Fig. 5N and O). Conversely, tRF3a-MetCAT knockdown reduced STAT1 stability. These results demonstrate that tRF3a-MetCAT modulates C5a expression by regulating the stability of its transcription factor STAT1.

### TRIM21 interacts with STAT1 and promotes its ubiquitin-mediated degradation

**Fig. 6.**
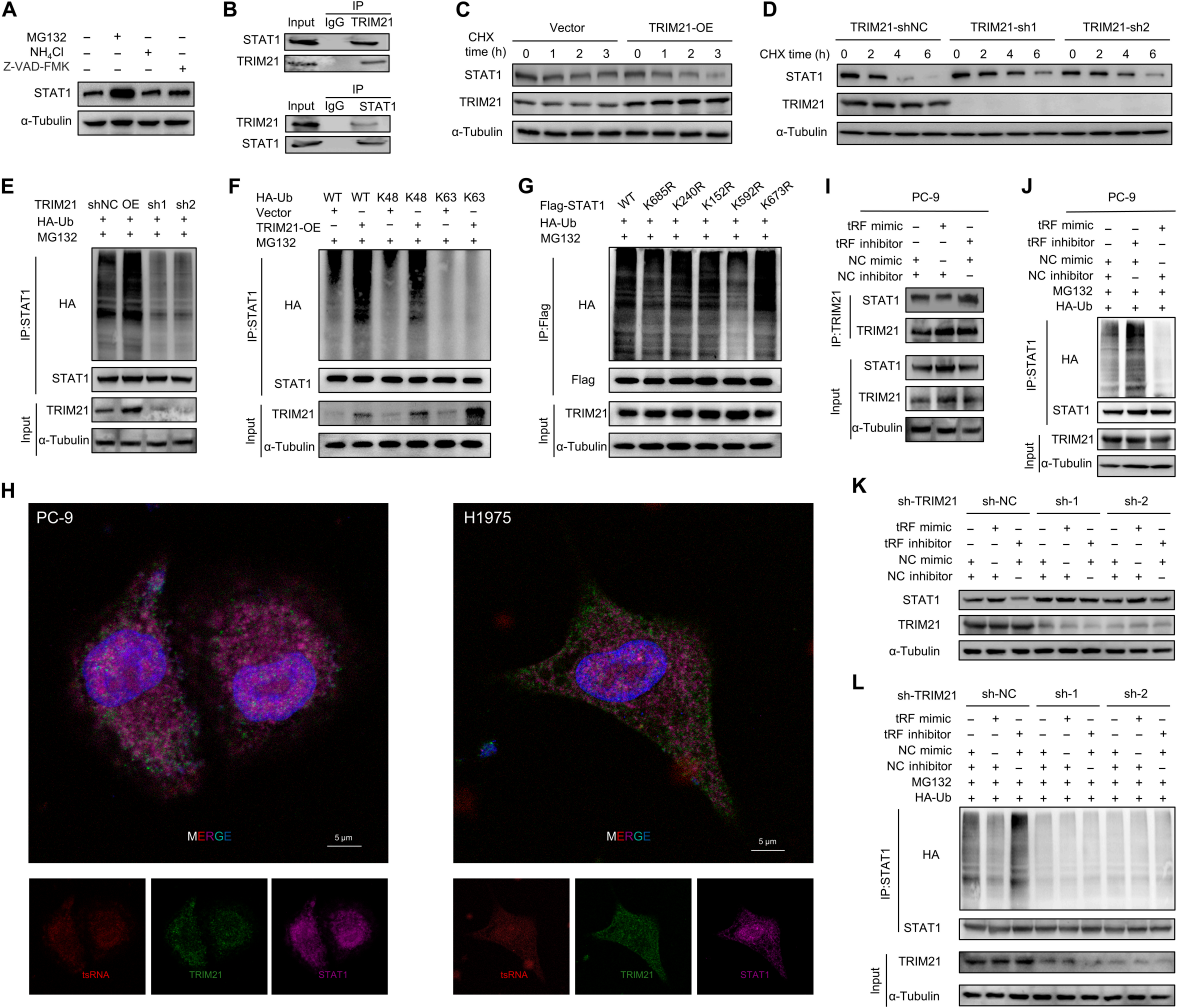
tRF3a-MetCAT regulates TRIM21-mediated ubiquitination of STAT1. (A) Western blot analysis of STAT1 expression in PC-9 cells treated with various inhibitors. (B) Co-IP analysis of the interaction between STAT1 and TRIM21 with anti-TRIM21 antibody (top) or with anti-STAT1 antibody (bottom) in PC-9 cell lines. (C) Half-life analysis of STAT1 in PC-9 cell lines overexpressing TRIM21 or vector, treated with CHX (40 μg/ml) for the indicated times. (D) Half-life analysis of STAT1 treated with CHX (40 μg/ml) for the indicated times in a TRIM21-shNC, TRIM21-sh1, or TRIM21-sh2 stable cell line. (E) Western blot analysis of STAT1 ubiquitination. PC-9 cells were transfected with the indicated plasmids or using indicated stable cell lines and were treated with MG132 (10 mM) for 6 h. (F) Western blot analysis of STAT1 ubiquitination after being transfected with HA-Ub mutants (WT, K48, and K63). (G) Western blot analysis of STAT1 ubiquitination after mutation of the top 5 predicted lysine sites to arginine. (H) The co-localization of STAT1 and TRIM21 with tRF3a-MetCAT was examined in PC-9 and H1975 cell lines using immunofluorescence. Scale bars: 5 μm. (I) Co-IP analysis of the interaction between STAT1 and TRIM21 in PC-9 cells overexpressing or inhibiting tRF3a-MetCAT. (J) Western blot analysis of STAT1 ubiquitination in PC-9 cells overexpressing or inhibiting tRF3a-MetCAT. (K) Western blot analysis of STAT1 protein levels in TRIM21-knockout or control PC-9 cells overexpressing or inhibiting tRF3a-MetCAT. (L) Western blot analysis of STAT1 ubiquitination in TRIM21-knockout or control PC-9 cells overexpressing or inhibiting tRF3a-MetCAT. Z-VAD-FMK, benzyloxycarbonyl-Val-Ala-Asp(OMe)-fluoromethylketone; HA-Ub, HA-tagged ubiquitin; K48/K63, ubiquitin with only lysine 48/63 retained.

TRIM21 suppresses C5a transcription, and STAT1, a transcription factor for C5, may interact with TRIM21. Therefore, we hypothesized that TRIM21 down-regulates C5a expression by promoting STAT1 degradation. To examine this assumption, we first studied the degradation pathway of STAT1. Treatment with the proteasome inhibitor MG132 markedly increased STAT1 levels, indicating that STAT1 was primarily degraded via the ubiquitin–proteasome pathway (Fig. 6A). Co-IP assays validated the interaction of TRIM21 with STAT1 within PC-9 cells (Fig. 6B). Moreover, TRIM21 overexpression significantly reduced the half-life of STAT1, whereas TRIM21 knockdown extended it (Fig. 6C and D and Fig. S5B and C). Cotransfection with ubiquitin-expressing plasmids further revealed that TRIM21 overexpression enhanced STAT1 ubiquitination, whereas TRIM21 knockdown reduced it (Fig. 6E). To determine the type of ubiquitin linkage, we transfected cells with ubiquitin mutants retaining only K48 or K63. The K48 mutant promoted STAT1 ubiquitination similarly to wild-type ubiquitin, while the K63 mutant did not, indicating that TRIM21 mediates K48-linked ubiquitination (Fig. 6F). To identify specific ubiquitination sites, we predicted candidate lysine residues using the GPS-Uber and mutated the top 5 sites (Fig. S5D) [28]. Mutation of lysine 592 (K592R) significantly reduced STAT1 ubiquitination, suggesting it as a key site (Fig. 6G), and overexpression of K592R STAT1 led to increased C5 mRNA levels compared to those of the wild type, indicating that ubiquitination at K592 negatively regulates C5 transcription (Fig. S5E). Collectively, these findings establish that TRIM21 acts as an E3 ubiquitin ligase for STAT1, thereby promoting its proteasomal degradation through K48-linked ubiquitination.

### tRF3a-MetCAT modulates the interaction between TRIM21 and STAT1

To further elucidate the relationship between tRF3a-MetCAT, TRIM21, and STAT1, immunofluorescence assays revealed co-localization and potential interactions among 3 proteins in EGFR-mutant LUAD cell lines (Fig. 6H). Co-IP experiments demonstrated that tRF3a-MetCAT overexpression reduced the interaction between STAT1 and TRIM21, resulting in decreased STAT1 ubiquitination and elevated STAT1 protein levels (Fig. 6I and J). Notably, the effect of tRF3a-MetCAT on STAT1 expression was abolished by TRIM21 knockdown (Fig. 6K). Consistently, tRF3a-MetCAT failed to alter STAT1 ubiquitination levels in TRIM21-deficient cells (Fig. 6L). These findings indicate that tRF3a-MetCAT interaction stabilizes STAT1 protein by interfering with TRIM21–STAT1 interaction, thereby reducing the TRIM21-mediated ubiquitination of STAT1.

### tRF3a-MetCAT impairs EGFR-TKI efficacy in vivo, while targeting C5 enhances the therapeutic response

**Fig. 7.**
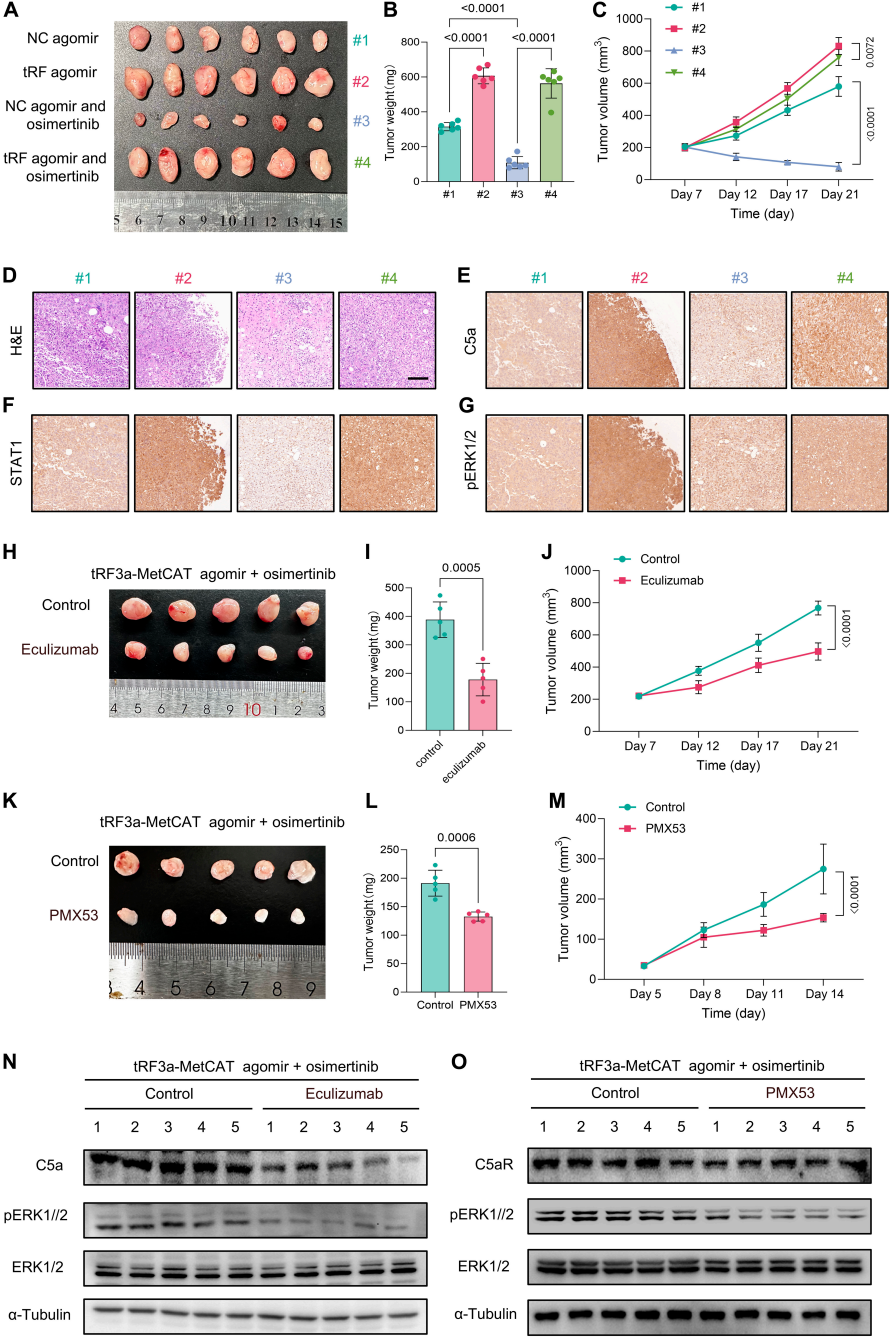
tRF3a-MetCAT overexpression impairs the therapeutic efficacy of EGFR-TKI in vivo. (A to C) Xenograft tumors were established by subcutaneous injection of control or tRF3a-MetCAT-overexpressing PC-9 cells into nude mice, followed by treatment with either osimertinib or vehicle. Representative tumor images are shown in (A); tumor weight (B) and tumor volume (C) were measured and analyzed (*N* = 5 mice per group). (D to G) Representative H&E staining (D) and immunohistochemistry (IHC) staining for C5a (E), STAT1 (F), and p-ERK1/2 (G) in tumor tissues from the groups described in (A). (H to J) Nude mice bearing subcutaneous PC-9 xenografts overexpressing tRF3a-MetCAT were treated with osimertinib alone or in combination with eculizumab. Representative tumor images are shown in (H); tumor weight (I) and tumor volume (J) were measured and analyzed (*N* = 5 mice per group). (K to M) Nude mice bearing subcutaneous PC-9 xenografts overexpressing tRF3a-MetCAT were treated with osimertinib alone or in combination with PMX53. Representative tumor images are shown in (K); tumor weight (L) and tumor volume (M) were measured and analyzed (*N* = 5 mice per group). (N) Western blot analysis of C5a and p-ERK1/2 levels in tumor tissues derived from tRF3a-MetCAT-overexpressing PC-9 xenografts under the indicated treatments. (O) Western blot analysis of C5aR and p-ERK1/2 levels in tumor tissues derived from tRF3a-MetCAT-overexpressing PC-9 xenografts under the indicated treatments. *P* values were determined by 1-way analysis of variance (ANOVA) (B) and 2-way ANOVA multiple comparisons at day 21 (C).

We assessed tRF3a-MetCAT’s function in EGFR-mutant LUAD through in vivo studies. In a subcutaneous xenograft mouse model, overexpression of tRF3a-MetCAT significantly attenuated the therapeutic effects of osimertinib (Fig. 7A to C). Immunohistochemistry (IHC) analysis confirmed that while osimertinib treatment reduced ERK1/2 phosphorylation, tumors overexpressing tRF3a-MetCAT exhibited elevated levels of STAT1, C5a, and phosphorylated ERK1/2, indicating the development of drug resistance (Fig. 7D to G). Based on the proposed mechanism, we hypothesized that targeting the C5a–C5aR axis could restore sensitivity to osimertinib in EGFR-mutant tumors with a high tRF3a-MetCAT expression. Eculizumab, a clinically approved C5 inhibitor, was used to test this hypothesis. Treatment of tRF3a-MetCAT-overexpressing PC-9 and H1975 cells with eculizumab resulted in reduced C5a levels in the culture supernatants (Fig. S6A). Furthermore, in vivo xenograft experiments demonstrated that eculizumab enhanced osimertinib efficacy in tumors overexpressing tRF3a-MetCAT, partially overcoming resistance (Fig. 7H to J). In addition, treatment with the C5aR antagonist PMX53 significantly enhanced EGFR-TKI efficacy (Fig. 7K to M). Western blot and IHC analyses revealed decreased levels of C5a and phosphorylated ERK1/2 after eculizumab treatment (Fig. 7N and Fig. S6B). A similar decrease in ERK1/2 phosphorylation was observed following PMX53 treatment, despite no significant change in C5aR expression levels (Fig. 7O and Fig. S6C). As a key component of the innate immune system, complement C5 may mediate the immunoregulatory effects of tRF3a-MetCAT within the tumor microenvironment. Analysis of the TIMER database revealed that C5 mRNA expression in LUAD is positively correlated with M2 macrophage infiltration and negatively correlated with M1 macrophage levels, suggesting that C5 may contribute to an immunosuppressive milieu (Fig. S7A) [29]. To further explore this possibility, we established an in vitro co-culture model using EGFR-mutant LUAD cells (PC-9) and macrophages derived from human peripheral blood mononuclear cell (PBMC)-isolated monocytes. Overexpression of tRF3a-MetCAT in tumor cells promoted the M2 polarization of macrophages, as indicated by up-regulated expression of *IL10* and *ARG1* (Fig. S7B and C). Treatment with a C5 or C5aR inhibitor attenuated this phenotype, reducing both M2 macrophage proportions and immunosuppressive gene expression. Notably, the effect was more pronounced with C5aR blockade, likely due to C5aR expression on macrophages themselves, further supporting the involvement of the C5–C5a–C5aR axis in tRF3a-MetCAT-mediated immune modulation.

**Fig. 8.**
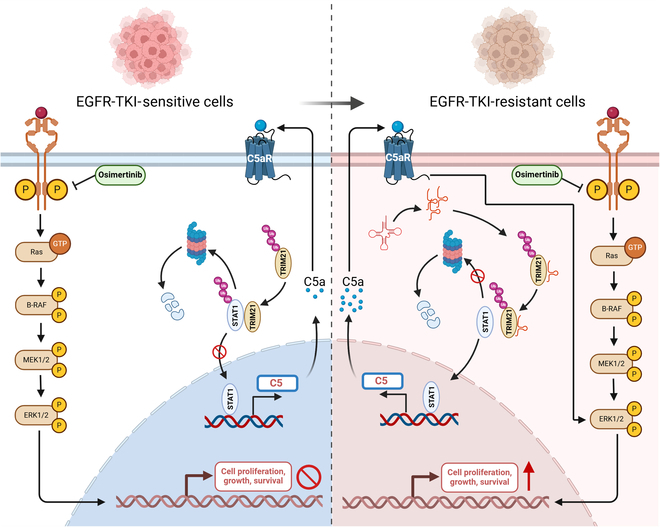
Schematic illustration showing that tRF3a-MetCAT promotes EGFR-targeted therapeutic resistance through the TRIM21–STAT1–C5a axis in LUAD. GTP, guanosine triphosphate.

Here, we found that elevated tRF3a-MetCAT levels correlated with osimertinib resistance in EGFR-mutant LUAD patients and that its high expression correlates with poor prognosis. Furthermore, tRF3a-MetCAT inhibited the interaction between TRIM21 and STAT1, thereby stabilizing STAT1 and promoting the transcription of downstream C5 and C5a. This, in turn, activates the C5a/C5aR/ERK pathway, ultimately resulting in osimertinib resistance both in vitro and in vivo (Fig. 8). Targeting the key molecule, C5a, in this resistance pathway can partially improve the efficacy of osimertinib treatment in vivo and alleviate resistance. Our research offers novel perspectives on the function of tsRNAs in LUAD-targeted therapy resistance and offers novel strategies to overcome targeted therapy resistance.

## Discussion

For LUAD patients harboring EGFR mutations, EGFR-TKIs have dramatically changed the treatment landscape, resulting in better therapeutic outcomes. However, the emergence of EGFR-TKI resistance remains a major clinical challenge, limiting their long-term efficacy [30]. Resistance mechanisms are not solely driven by secondary EGFR mutations but also involve a range of EGFR-independent pathways, including bypass signaling activation, epigenetic alterations, and remodeling of the tumor microenvironment. These mechanisms contribute to drug escape and limit the efficacy of targeted therapies [31]. For example, mesenchymal–epithelial transition factor (MET) amplification can mediate resistance by bypassing EGFR signaling and reactivating the downstream mitogen-activated protein kinase (MAPK) and phosphoinositide 3-kinase (PI3K) pathways, thereby promoting tumor growth [32]. Similarly, human epidermal growth factor receptor 2 (HER2) mutations may independently activate downstream EGFR signaling. Genetic changes like PIK3CA mutations or PTEN deletion can amplify PI3K pathway activity, driving resistance in about 5% to 12% of patients on third-generation EGFR-TKIs [33]. Thus, inhibitors targeting these bypass pathways are being explored as key strategies to overcome EGFR-TKI resistance [34]. EGFR activates a signaling cascade involving RAS/RAF/MEK, ultimately leading to ERK1/2 phosphorylation and downstream transcriptional activation, which promotes tumor cell proliferation and metastasis [35–40]. Notably, the resistance mechanism mediated by tRF3a-MetCAT shares conceptual similarities with known EGFR-TKI bypass pathways such as HER2 mutations and MET amplification, in that all 3 converge on sustained activation of downstream signaling pathways, particularly ERK and PI3K/AKT, to promote drug resistance. However, the mechanistic routes are distinct. While HER2 mutations and MET amplification activate downstream signaling through receptor tyrosine kinase dimerization or cross talk with HER3, tRF3a-MetCAT modulates resistance via the C5a/C5aR axis, leading directly to ERK phosphorylation. Importantly, this mode of action appears to be independent of HER2 or MET status, suggesting that tRF3a-MetCAT represents a parallel and nonredundant bypass route. Our results underscore the crucial bypass activation is in the development of resistance, indicating that a multipronged treatment approach targeting various pathways could be a promising avenue for overcoming such challenges down the line. Whether tRF3a-MetCAT might also cooperate with or modulate HER2- or MET-driven resistance remains an open question and merits further investigation. This possibility raises the therapeutic potential of targeting C5a/C5aR signaling as a broader strategy to overcome diverse resistance mechanisms.

tRNAs constitute a category of noncoding RNAs vital for the process of protein production. A typical tRNA molecule comprises 76 nucleotides and folds into a stable cloverleaf shape. Under cellular stress conditions, tRNAs can be cleaved by specific endonucleases to generate fragments of varying lengths, collectively known as tsRNAs. As high-throughput sequencing evolves and interest in noncoding RNAs increases, accumulating evidence has revealed that tsRNAs are precisely regulated small RNAs. They are widely expressed across tissues and cell types and exhibit strong tissue, disease, and temporal specificity [41]. Importantly, tsRNAs circulate throughout bodily fluids—including urine, saliva, and semen—making them promising candidates for noninvasive diagnostic tools. Their presence could revolutionize early cancer detection, treatment monitoring, and personalized medicine by offering a simple yet effective way to track disease progression [42]. tsRNAs engage in numerous processes, such as stress responses, stem cell maturation, ribosome biogenesis, and tumorigenesis [43–48]. They regulate transposon silencing, protein translation, and epigenetic modulation. Many tsRNAs are differentially expressed in cancer cells compared with those in normal cells and are rising as pivotal contributors to the onset and advancement of cancer. In breast cancer, specific tsRNAs can displace YBX1 from the 3′ untranslated regions of target mRNAs, thereby diminishing the stability of various oncogenic transcripts and suppressing tumor cell proliferation, invasion, and metastasis [49]. In hepatocellular carcinoma, LeuCAG3 tsRNA promoted translation by binding to ribosomal protein mRNAs [50]. In colorectal cancer, 5′tiRNA-Gly-GCC boosts 5-fluorouracil resistance via SPIB targeting and JAK1/STAT6 modulation [51], while 5′tiRNA-His-GTG enhances tumor progression by down-regulating LATS2 mRNA translation [52]. Despite the emerging importance of tsRNAs in cancer biology, their role in EGFR-TKI resistance has not been elucidated. This research revealed a new tsRNA, tRF3a-MetCAT, originating from tRNA-MetCAT, excessively present in osimertinib-resistant LUAD organoids and linked strongly to unfavorable outcomes for LUAD patients, revealing a novel EGFR-independent resistance mechanism. Interestingly, tsRNA production is typically triggered under cellular stress. We observed that tRF3a-MetCAT levels increased progressively with prolonged osimertinib treatment (Fig. 1), suggesting that osimertinib itself may act as a stressor that induces tsRNA generation. Although we did not explore the upstream mechanisms of tRF3a-MetCAT induction in this study, previous research has shown that various forms of cellular stress, including ultraviolet radiation, heat shock, exposure to heavy metals, and alterations in oxidative stress levels, can trigger tRNA cleavage through stress-responsive nucleases such as angiogenin [9,53]. Osimertinib treatment may induce such stress by altering intracellular oxidative stress levels, potentially promoting the biogenesis of tRF3a-MetCAT. This possibility highlights the need for future studies to clarify the precise mechanisms linking EGFR-TKI treatment to tsRNA generation. Exploring strategies to reduce such stress responses would potentially offer novel approaches for improving the durable efficacy of EGFR-TKI treatments. Given its inducibility and potential stability in circulation, tRF3a-MetCAT may also serve as a noninvasive biomarker for monitoring resistance development during EGFR-TKI therapy.

Interestingly, TRIM21 contains a PRY–SPRY domain at its C-terminus, a structural feature commonly found in the TRIM protein family. While TRIM21 has not been classically recognized as an RNA-binding protein, recent studies have suggested that the PRY–SPRY domain possesses intrinsic RNA-binding potential. Notably, TRIM25, another TRIM family member, has been shown to directly bind RNA through its PRY–SPRY domain, and this RNA-binding function is retained even when the domain is replaced by the homologous sequence from TRIM21 [54]. In this study, we demonstrated a direct interaction between TRIM21 and tRF3a-MetCAT, and this binding is likely mediated by the PRY–SPRY domain. These findings provide new evidence supporting the RNA-binding potential of the PRY–SPRY domain and lay the groundwork for future studies exploring its role in RNA recognition.

The complement system is closely linked to inflammatory responses, and understanding how tumor-associated inflammation is regulated may enhance the efficacy of cancer therapy [55–58]. Key complement components like C3a and C5a attract neutrophils, monocytes, and macrophages to inflamed tissues through strong chemotactic activity [59]. Consequently, the complement system critically influences the tumor immune microenvironment. For instance, the C5a/C5aR signaling axis can recruit immunosuppressive cells, including tumor-associated macrophages and regulatory T cells. This process fosters an immune-suppressive environment that ultimately fuels tumor growth [20,60]. Beyond its immunomodulatory roles, the C5a/C5aR axis can directly affect tumor cells by promoting oncogenic signaling [61–63]. One major mechanism of targeted therapy resistance is the activation of bypass signaling pathways [64]. C5a/C5aR has been shown to activate several critical pathways, including PI3K/Akt, nuclear factor-κB, and MAPK, which collectively support tumor cell proliferation and survival [65,66]. Given these multifaceted roles, targeting the C5a/C5aR pathway shows potential in countering drug resistance and suppressing tumor progression. Although several inhibitors targeting the C5a/C5aR axis are already available, such as eculizumab (which targets C5a) and PMX53 (a C5aR antagonist), their current clinical use is restricted to immune-related conditions such as paroxysmal nocturnal hemoglobinuria and antineutrophil-cytoplasmic-antibody-associated vasculitis [67,68]. However, they are yet to be widely used in oncology. In this study, we discovered that complement component C5a contributes to EGFR-TKI resistance in LUAD by activating the ERK pathway. Although eculizumab does not directly bind or neutralize C5a, it prevents the cleavage of C5 into C5a and C5b by inhibiting the C5 convertase complex. Therefore, in our tumor model, eculizumab indirectly reduces the generation of C5a, thereby limiting downstream complement activation and C5a-mediated immunosuppressive effects in the tumor microenvironment (Fig. 7). These results indicate that targeting the complement pathway, particularly the C5a/C5aR axis, could represent a new strategy to overcome resistance in EGFR-mutant LUAD, and offer a new direction for future cancer immunotherapy strategies.

Here, we found that elevated tRF3a-MetCAT levels are linked to osimertinib resistance and worse outcomes in EGFR-mutant LUAD patients. Furthermore, we found that tRF3a-MetCAT inhibits the interaction between TRIM21 and STAT1, thereby stabilizing STAT1 and promoting the transcription of downstream C5 and C5a expression. This, in turn, activates the C5a–C5aR–ERK pathway, ultimately resulting in osimertinib resistance both in vitro and in vivo. Targeting the key molecule C5a in this resistance pathway can partially improve the efficacy of osimertinib treatment in vivo and alleviate resistance. Our research reveals novel findings on tsRNA’s involvement in LUAD treatment resistance and offers novel strategies for overcoming targeted therapy resistance.

## Methods

### Patient specimens and ethics statement

The patients’ specimens used for organoids and ISH staining were obtained from patients being treated at Zhongshan Hospital, Fudan University. Microarray specimens were sourced from patients who had complete surgical excision at Zhongshan Hospital, Fudan University, in 2015, with their clinicopathologic characteristics outlined in Table S1. Prior to surgery, informed consent was provided by all participants for their clinical data usage, and the research was authorized by Zhongshan Hospital, Fudan University’s Ethics Committee (No. B2021-128).

### Mouse subcutaneous tumor model and ethics statement

Male nude mice, 5 to 6 weeks old, received subcutaneous transplants of PC-9 cells (1 ∗ 10^6^) into their right back flanks (GemPharmatech, Jiangsu); 50 nmol of tRF3a-MetCAT agomir/antagomir or control agomir/antagomir was injected into the implanted tumor every 3 d (RiboBio, Guangzhou; Table S2). Animal studies obtained approval from Zhongshan Hospital’s Ethics Committee (No. 2023-220).

### Organoid culture and drug resistance induction

Following the guidelines provided by the manufacturer, human EGFR-mutant LUAD tissues were meticulously dissected into individual cells using Tumor Dissociation Kit (Miltenyi Biotec). These solitary cells were then embedded within a Matrigel matrix (Corning) and nurtured in an organoid-specific culture medium, Dulbecco’s modified Eagle medium (DMEM)/F12 supplemented with B27, N2, EGF, FGF10, Noggin, and R-spondin1. The medium was changed every 3 d, and organoids were passaged every 10 to 14 d. To induce drug resistance, organoids were exposed to ascending levels of osimertinib (from 10 to 200 nM) over a 2-month period.

### Cell culture

PC-9 (Research Resource Identifier [RRID]: CVCL_B260), H1975 (RRID: CVCL_1511), Beas-2B (RRID: CVCL_0168), and A549 cells (RRID: CVCL_0023) were sourced from the American Type Culture Collection and maintained in DMEM with 10% fetal bovine serum and 1% penicillin/streptomycin. The cells were incubated at 37 °C with 5% CO_2_. Prior to experimentation, all cell lines underwent routine mycoplasma screening to ensure contamination-free conditions.

### Transfection and lentiviral infection

Cells were plated into 6-well dishes and treated with Lipofectamine 8000 (Beyotime, C0533) as per the manufacturer’s guidelines when they reached 60% to 70% cell density. For lentiviral transduction, cells were plated into 6-well plates and transduced with shRNA-expressing or control lentiviruses (GeneChem, Shanghai) using polybrene. After 24 h, the medium was refreshed, and puromycin-resistant cells were isolated over 5 to 7 d. Efficiency was confirmed by immunoblotting. The sequences are listed in Table S2.

### RT-qPCR analysis

RNA extraction was carried out with RNAsimple Total RNA Kit (TIANGEN), and the isolated RNA was then converted to complementary DNA (cDNA) using cDNA Synthesis SuperMix (YEASEN). qPCR was then executed on a LightCycler 480II system from Roche, with reaction volumes of 10 μl containing the cDNA, primers, and Hieff UNICON qPCR SYBR Green Master Mix (YEASEN). To measure the level of tsRNA, the Bulge-Loop miRNA reverse transcription qPCR (RT-qPCR) kit (RiboBio) was utilized. The ΔΔCt method was applied to determine relative gene expression in relation to *ACTB* or U6. The primers for the targeted tsRNA and genes are detailed in Table S2.

### RNA pull-down assay and silver staining

A biotin-labeled tRF3a-MetCAT probe was designed and synthesized using standard RNA synthesis methods, labeling the 3′ end with biotin (Genomeditech, Shanghai). Around 20 million cells were lysed in a buffer solution supplemented with 80 U/ml RNasin Plus RNase Inhibitor (Promega). The resulting cell lysate was then mixed with a biotin-labeled RNA probe and incubated for 4 h. Following this, streptavidin-coated agarose beads (Invitrogen, Carlsbad, USA) were added, and the mixture was gently rotated at 4 °C for an additional 2 h. The samples were meticulously cleaned 4 to 5 times to eliminate any unwanted proteins. Next, the RNA and protein complexes were extracted by boiling them in a sodium dodecyl sulfate loading buffer, followed by separation through sodium dodecyl sulfate–polyacrylamide gel electrophoresis. The gels were then set in a solution of 40% methanol and 10% acetic acid, treated with 0.02% sodium thiosulfate to enhance sensitivity, and stained with a 0.1% to 0.2% silver nitrate solution for dark, 20-min incubation. The bands emerged after they were developed in a 2% sodium carbonate bath with a touch of 0.04% formaldehyde. To halt the process, 1% acetic acid was used. The clear bands were carefully cut out, destained, and subjected to trypsin digestion. The resulting peptides were then analyzed using liquid chromatography–tandem mass spectrometry to identify the proteins.

### Mass spectrometry

The peptide samples, after desalting, were examined using a high-resolution Q Exactive HF-X Hybrid Quadrupole Orbitrap mass spectrometer (Thermo Scientific). The acquired mass spectrometry data were cross-referenced with a curated UniProt database limited to *Homo sapiens* entries, followed by peptide and protein identification through the PEAKS Online Xpro software. This analytical work was performed at the Core Facility of Shanghai Medical College, Fudan University.

### RIP assay

Magna RIP RNA-Binding Protein IP Kit (Millipore) was employed for RIP assay, adhering to the provided protocol. Cells were lysed in RIP buffer and incubated overnight at 4 °C with either 5 μg of TRIM21 antibody or immunoglobulin G (IgG) control. RNA was then isolated and analyzed via RT-qPCR.

### IHC and ISH

Tumor tissues were preserved in a 4% paraformaldehyde solution, embedded in paraffin, and sliced into thin sections. To uncover the antigens, the samples underwent antigen retrieval, after which any circulating peroxidase activity was blocked using a 3% hydrogen peroxide solution. Slides were immersed in Immunol Staining Blocking Solution for 30 min and then, following this, held at 4 °C in a primary antibody solution targeting C5a, STAT1, and p-ERK1/2 throughout the night. Following the washing step, the slides were treated with a secondary antibody for half an hour at room temperature. The reaction was then visualized using 3,3′-diaminobenzidine substrate before the slides were coverslipped. ISH analysis involved ordering the probe and FISH Kit (Boster Biological Technology).

### Cell growth, colony formation, migration, and invasion assays

Cell growth was measured with Cell Counting Kit-8 (Meilunbio). Cells were plated at 1,000 per well in 96-well plates and incubated for 0, 24, 48, 72, and 96 h. IC_50_ values were determined using nonlinear regression (4-parameter logistic curve fitting). Colony formation was assessed by plating 500 cells into 6-well plates and culturing for 14 d. Colonies were methanol-fixed, crystal violet-stained, and measured. Migration assays were performed using wound healing. Confluent cells in 6-well plates were braded using a sterile pipette tip and imaged at 12 and 24 h to assess wound closure. For invasion assays, cells cultured in serum-free medium were placed into upper chambers coated with Matrigel (Corning), with serum-containing medium in the lower chambers. After 48 to 72 h, invaded cells were fixed, stained, and counted.

### Western blot and Co-IP

Cells were rinsed with cold phosphate-buffered saline and then lysed using a cell lysis buffer infused with 1% phenylmethanesulfonyl fluoride and phosphatase inhibitors. For Co-IP, supernatants were mixed overnight with antibodies and protein A/G beads (Santa Cruz) at 4 °C. Beads underwent washing, boiling in loading buffer, and Western blot analysis. The antibodies used followed the manufacturer’s guidelines, as detailed in Table S3.

### Enzyme-linked immunosorbent assay

Supernatants from the cell cultures were harvested, and the concentration of C5a was determined through a standardized enzyme-linked immunosorbent assay procedure supplied by R&D Systems (DY2037). Following the protocol, a microplate reader measured absorbance at 450 nm.

### Immunofluorescence

Cells were fixed in 4% paraformaldehyde, blocked, and exposed to primary antibodies overnight at 4 °C, after which they were stained with fluorescent secondary antibodies for 2 h at RT. Cy3-labeled tRF3a-MetCAT probe (RiboBio, Guangzhou) was used for tsRNA staining. Microscopic images were captured using Leica TCS SP5, with samples mounted in antifade solution.

### Protein half-life assays

Cells were plated into 6-well dishes and exposed to cycloheximide (40 μg/ml) across specified time intervals, with or without prior plasmid transfection. Proteins were extracted at each time point, analyzed by Western blot, and quantified by ImageJ.

### Dual-luciferase reporter assay

The C5 predicted promoter or mutant promoter was inserted into a pGL4.10 vector. The C5 promoter construct was introduced into cells alongside a pGL4.75 plasmid encoding *Renilla* luciferase as an internal control. Using a commercially available dual-luciferase reporter assay kit (Vazyme), we quantified both firefly and *Renilla* luciferase activities following the supplier’s protocol. To normalize the data, we determined relative luciferase activity by dividing the firefly luminescence values by the corresponding *Renilla* readings.

### Microscale thermophoresis

The interactions between TRIM21 and tRF3a-MetCAT were measured by MST using the NanoTemper Monolith NT.115 instrument. Recombinant TRIM21 protein served as a target and was fluorescently labeled with Monolith Protein Labeling Kit RED-NTA (MO-L018, NanoTemper Technologies). tRF3a-MetCAT was employed as the ligand. For each assay, the labeled protein (50 nM) was incubated with varying concentrations (400 to 0.0061 μM) of the ligand in the MST buffer (137 mM NaCl, 2.68 mM KCl, 8.1 mM sodium phosphate buffer, 1.76 mM KH_2_PO_4_, pH 7.4, 0.05% Tween-20) at room temperature for 15 min. The samples were loaded into NT.115 standard capillaries. Analyses were performed at 25 °C, 100% excitation power, and 40% MST power. Apparent *K*_D_ values were calculated using MO Affinity Analysis v2.3. Data of at least 3 independently pipetted measurements were analyzed, and *K*_D_ values are expressed as mean ± standard error of the mean.

### ChIP assay

ChIP analysis was executed with BersinBio Chromatin Immunoprecipitation Kit, adhering to the provided protocol. In brief, 1 × 10^7^ PC-9 and H1975 cells were treated with 1% formaldehyde for cross-linking and then lysed and processed into 200- to 750-bp chromatin fragments via sonication. Chromatin was immunoprecipitated overnight at 4 °C with 5 μg of anti-STAT1 or control IgG and protein A/G beads. After reverse cross-linking and DNA purification, target enrichment was analyzed by PCR. Primers for the target region are listed in Table S2.

### Macrophage co-culture and flow cytometry

Human PBMCs were isolated by Ficoll density gradient centrifugation. Monocytes were negatively selected using CD14 magnetic beads (STEMCELL) and cultured in RPMI 1640 with 10% fetal bovine serum and 50 ng/ml M-CSF for 7 d to generate M0 macrophages. For indirect co-culture, M0 macrophages were seeded in the lower chamber of a 0.4-μm Transwell system, with PC-9 cells (NC or tRF3a-MetCAT overexpression) in the upper chamber. Where indicated, eculizumab or PMX53 was added. After 48 h, macrophages were harvested; stained for CD45, CD68, CD206, and CD80; and analyzed by flow cytometry.

### Arraystar small RNA microarray and RNA sequencing

To identify tsRNA levels in osimertinib-resistant and osimertinib-sensitive organoids, an Arraystar small RNA microarray assay was performed by Aksomics (Shanghai, China). Poly(A)-tailed mRNA was purified using oligo(dT) beads and then fragmented to generate cDNA templates. After end repair, adaptor ligation, and size selection, libraries were constructed and PCR-amplified. Sequencing was performed on Illumina HiSeq/NovaSeq or MGI2000 platforms using 2×150 bp paired-end reads. The sequencing data underwent initial quality control using Cutadapt (version 1.9.1) to eliminate substandard reads and adapter sequences. Following this cleanup, the high-quality reads were mapped to the GRCh38 human reference genome employing Hisat2 (version 2.2.1). Finally, transcript quantification was performed with HT-seq (version 0.6.1) to generate gene expression counts. Differential gene expression was analyzed using DESeq2 (v1.38.3). GSEA and single-sample GSEA were performed with GSEABase (v1.60.0) using gene sets from MSigDB (HALLMARK, REACTOME, and WikiPathways). Library prep, sequencing, and initial analysis were conducted by Genewiz, Inc.

### Statistical analysis

The data were meticulously examined with the GraphPad Prism software suite, version 8, developed by GraphPad Software Inc. in La Jolla, CA, USA. The results are displayed as average values plus or minus the standard deviation. To determine any variations in continuous data across different groups, the Student unpaired *t* test, 1-way analysis of variance (ANOVA), or 2-way ANOVA method was employed. For categorical data, the chi-square test was utilized for comparison. Univariate Cox regression was used for survival analysis. Findings with *P* values below 0.05 were deemed to hold statistical significance.

## Data Availability

Any additional information required to reanalyze the data reported in this paper is available from the lead contact upon request.
