# Supplementary material for: tRF3a-MetCAT Promotes EGFR-Targeted Therapeutic Resistance through the TRIM21–STAT1–C5a Axis in Lung Adenocarcinoma
Source: Research (Wash D C). 2025 Oct 7;8:0911. doi: 10.34133/research.0911 (PMC12501432; doi:10.34133/research.0911)
Supplement: Supplementary 1 — Figs. S1 to S7 Tables S1 to S4 [file research.0911.f0911.docx]

**Supplementary Materials**

tRF3a-MetCAT promotes EGFR-targeted therapeutic resistance through the TRIM21-STAT1-C5a axis in lung adenocarcinoma

Yuchen Zhang^1†^, Pingjing Zhou^1†^, Yifan Guo^2^, Hongyu Zhang^1^, Jie Gu^1^*，Di Ge^1^* and Guangyin Zhao^2*^

^1^Department of Thoracic Surgery, Zhongshan Hospital, Fudan University, Shanghai 200032, China

^2^Department of Thoracic Surgery, Shanghai Geriatric Medical Center, Shanghai 201104, China

*Address correspondence to : gu.jie3@zs-hospital.sh.cn(J.G.), ge.di@zs-hospital.sh.cn(D.G.), zhao.guangyin@zsgmc.sh.cn(G.Z)

^†^These authors contributed equally to this work.


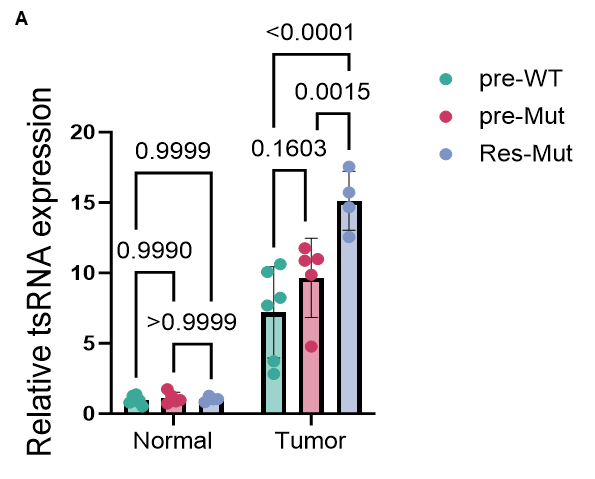


**FigS1. tRF3a-MetCAT is highly expressed in tumor tissues compared to paired normal tissues** (A) qRT-PCR analysis of tRF3a-MetCAT expression in different tissues.

**
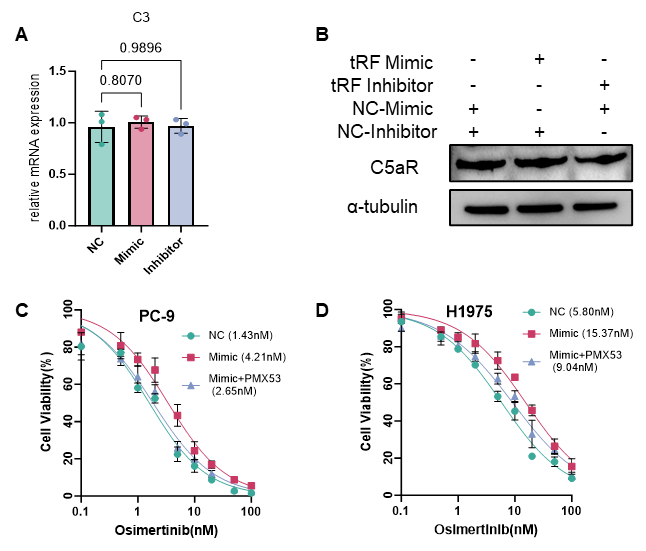
Fig.S2. tRF3a-MetCAT does not affect C3 transcription or C5aR expression** (A). qRT-PCR analysis of C3 mRNA expression levels in PC-9 cells overexpression or inhibition of tRF3a-MetCAT. (B) Western blot analysis of C5aR levels in PC-9 cells overexpression or inhibition of tRF3a-MetCAT. (C, D) Relative cell viability of indicated PC-9(C) and H1975(D) cells treated with Osimertinib for 5 days


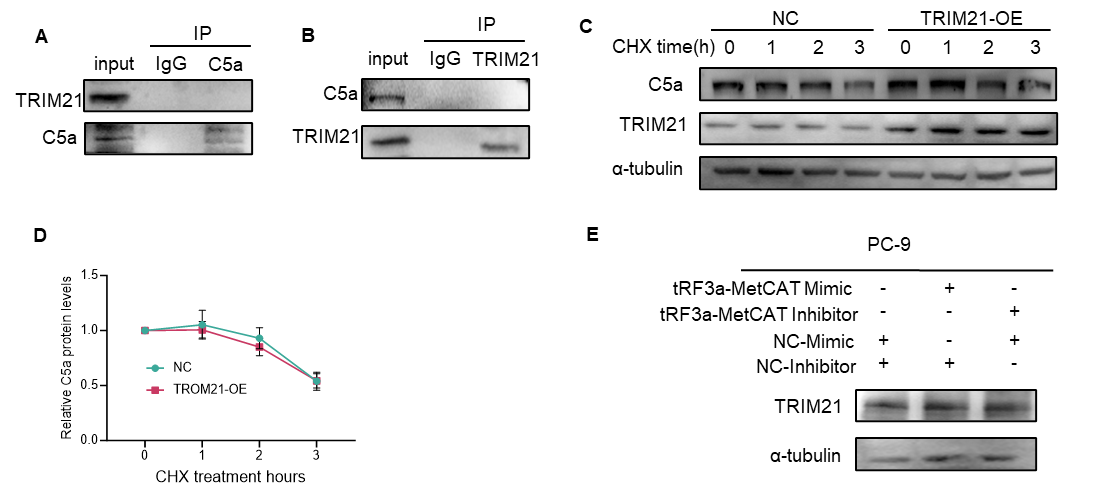


**FigS3. TRIM21 is not the E3 ubiquitin ligase of C5a and tRF3a-metCAT does not affect TRIM21 expression** (A,B) Co-IP analysis of the interaction between C5a and TRIM21 with anti-C5a antibody(A) or with anti-TRIM21 antibody(B) in PC-9 cell lines. (C, D) Half-life analysis of C5a after over-expressed TRIM21 (TRIM21-OE) or control (NC) treated with CHX (40µg/ml) for the indicated times in PC-9 cell lines (C). The quantifications were shown on (D). (E) Western blot analysis of TRIM21 expression in PC-9 cells with overexpression or inhibition of tRF3a-MetCAT.


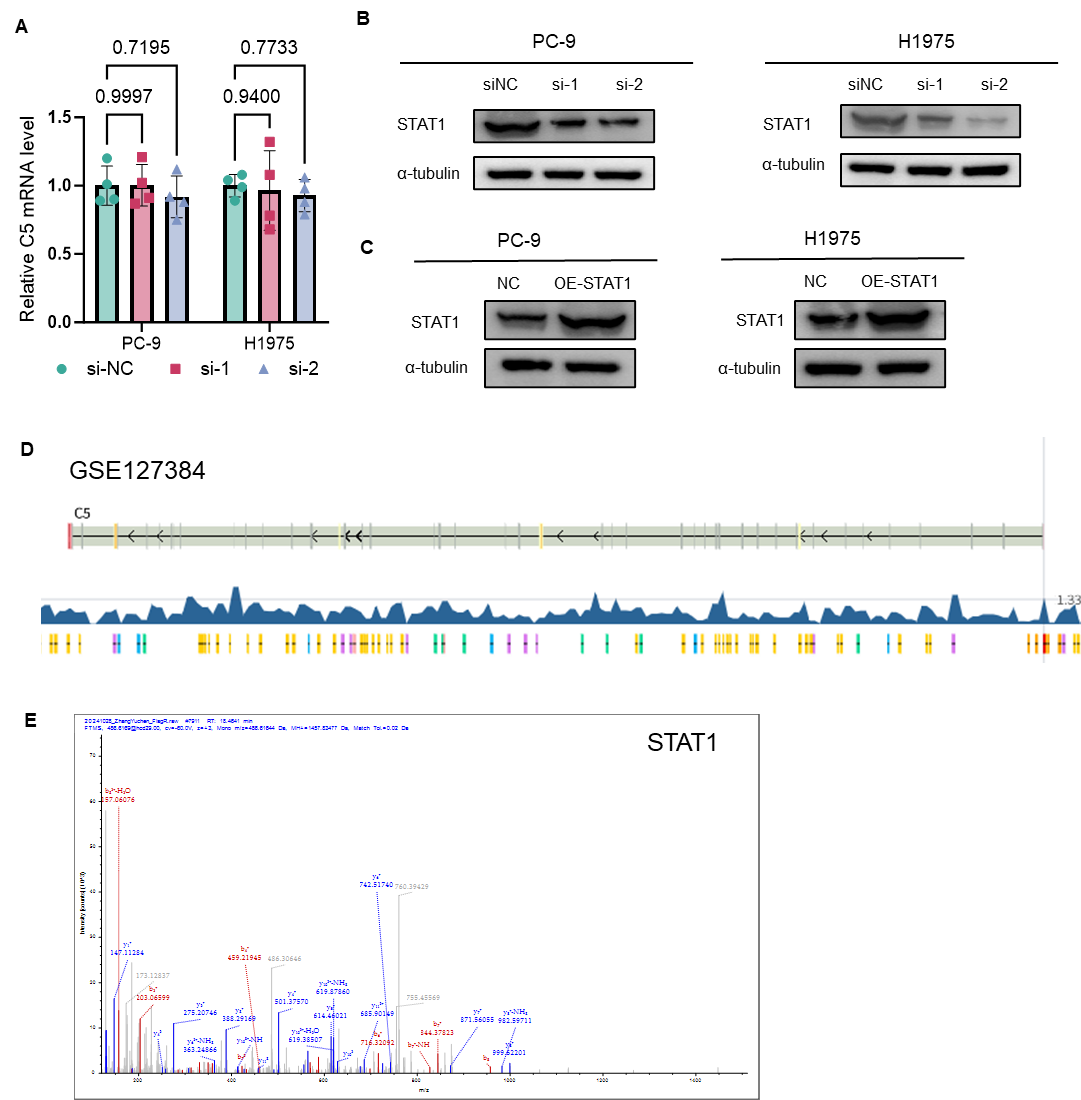


**FigS4. STAT1 is a transcription factor of C5, rather than HDAC2.** (A) qRT-PCR analysis of C5 mRNA levels following HDAC2 knockdown. (B) Western blot analysis of STAT1 expression in control (si-NC) and STAT1-knockdown (si-1, si-2) PC-9 and H1975 cell lines. (C) Western blot analysis of STAT1 expression in control (NC) and STAT1-overexpressing (STAT1-OE) PC-9 and H1975 cell lines. (D) Public ChIP-seq datasets suggest that STAT1 binds to the promoter region of C5. (E)Mass spectrometry confirms the interaction between STAT1 and TRIM21.

**
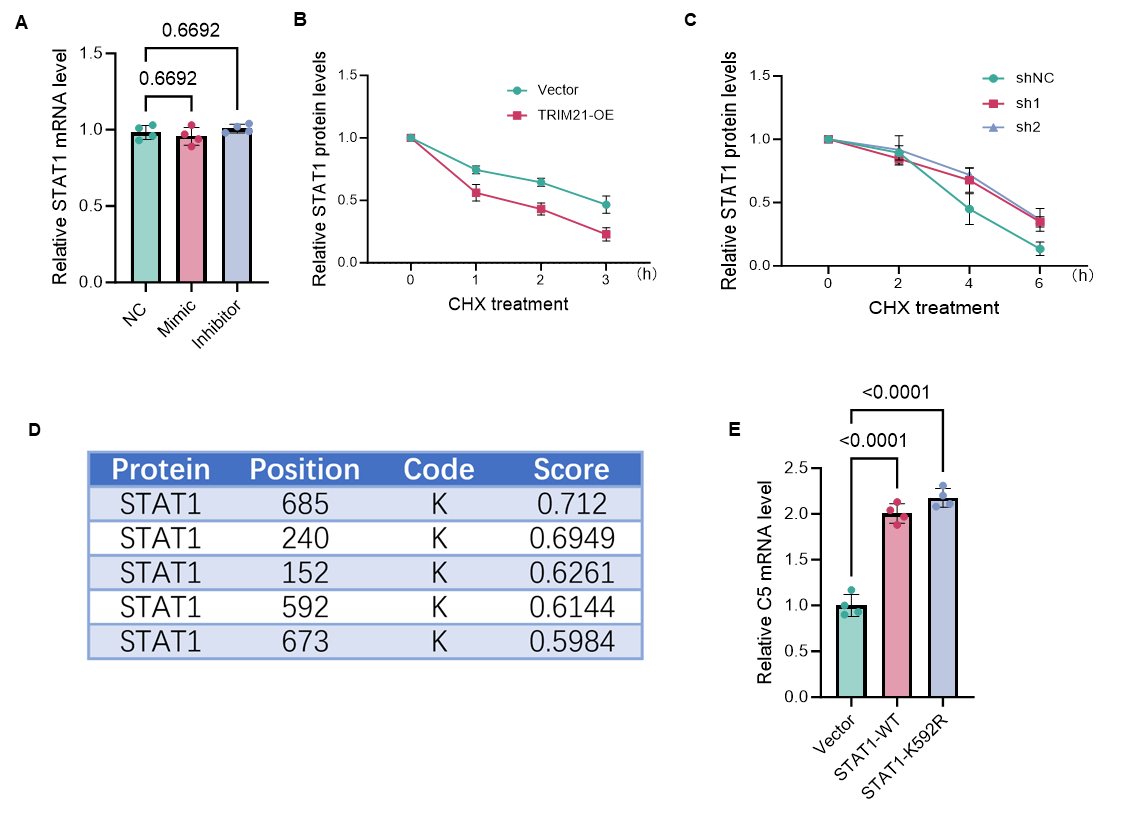
Fig S5. tRF3a-MetCAT reduces STAT1 protein stability through TRIM21, without altering STAT1 mRNA levels.** (A) qRT-PCR analysis of STAT1 mRNA expression levels in PC-9 cells with overexpression or inhibition of tRF3a-MetCAT. (B) The quantifications of half-life analysis of STAT1 related to Fig.6C. (C) The quantifications of half-life analysis of STAT1 related to Fig.6D. (D) Prediction of STAT1 ubiquitination sites. (E) qRT-PCR analysis of C5 mRNA expression levels in PC-9 cells with overexpression wild-type STAT1 or K592R mutant STAT1.

**
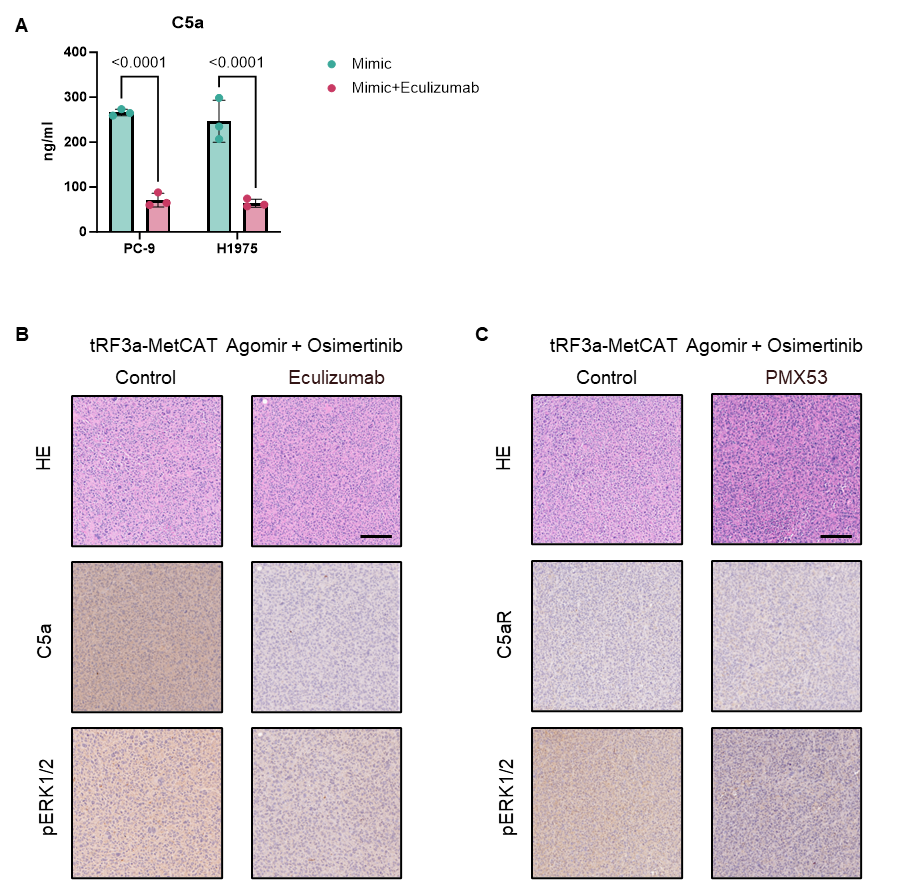
FigS6. Inhibition of C5a–C5aR signaling restores EGFR-TKI sensitivity in tRF3a-MetCAT–high tumors.** (A) ELISA analysis of C5a levels in the supernatant of PC-9 and H1975 cells overexpression tRF3a-MetCAT after treatment with eculizumab. (B) Representative H&E and IHC images showing C5a and p-ERK1/2 expression in tumor tissues from the treatment groups described in Fig.7H. (C) Representative H&E and IHC images showing C5aR and p-ERK1/2 expression in tumor tissues from the treatment groups described in Fig.7K.


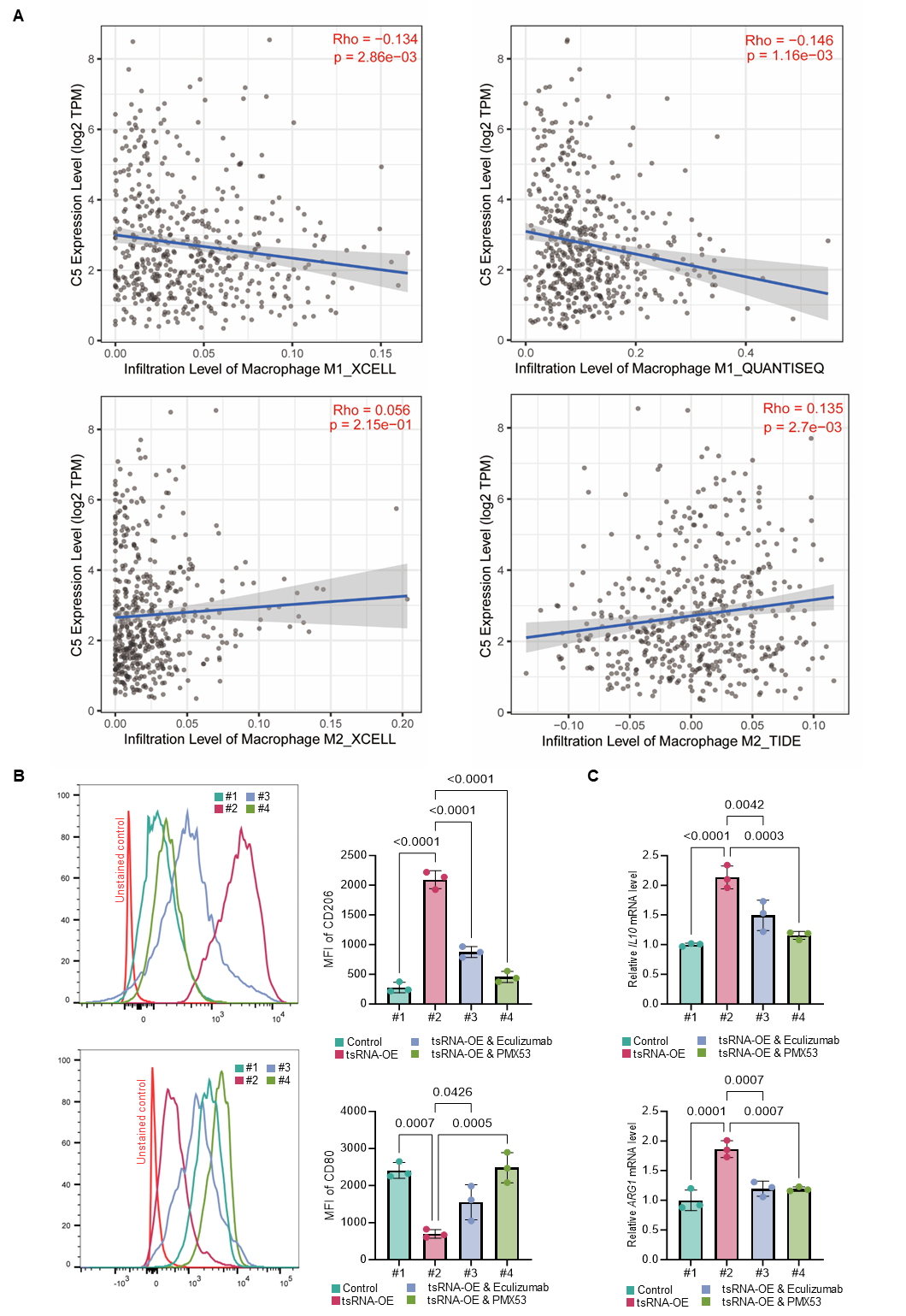


**FigS7. tRF3a-MetCAT promotes M2 macrophage polarization via C5 in the tumor microenvironment.** (A) Correlation between C5 mRNA expression and M1/M2 macrophage infiltration in LUAD. (B) Flow cytometric analysis of CD206 and CD80 expression in macrophages under different treatments. Quantification is shown on the right. (C) qRT-PCR analysis of *IL10/ARG1* mRNA expression levels in macrophages under different treatments

Table S1. Relation between tRF3a-MetCAT expression and clinical characteristics of 120 LUAD patients by immunohistochemistry with tissues microarray


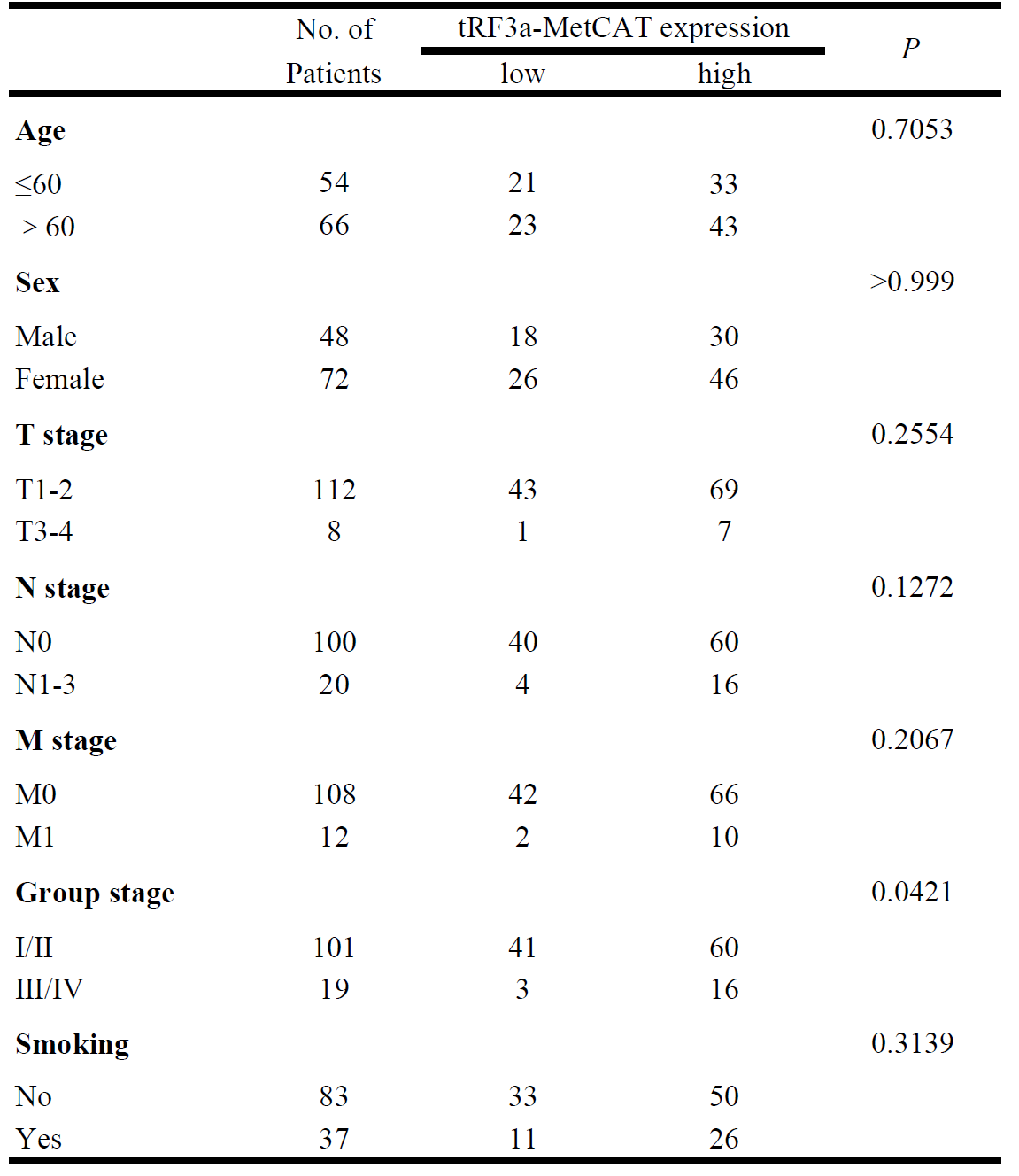


Table S2. Details of primers and shRNA sequences used in this research

| Details of primers and shRNA sequences used in this research | |
| --- | --- |
| qRT-PCR | |
| C3 | F:AAGTCGGCAAGTACCCCAAG |
|  | R:TGTAGTTGCAGCAGTCCAGG |
| C5 | F:TTCGCCTTCGGATTGCGTT |
|  | R:TGAACATGGCCTGAGGAGTA |
| STAT1 | F:CAGCTTGACTCAAAATTCCTGGA |
|  | R:TGAAGATTACGCTTGCTTTTCCT |
| IL10 | F:GAGAACAGCTGCACCCACTT |
|  | R:GGCAACCCAGGTAACCCTTA |
| ARG1 | F:GGGTTGACTGACTGGAGAGC |
|  | R:CGTGGCTGTCCCTTTGAGAA |
| ACTIN | F:TCGTGCGTGACATTAAGGAGAAGC |
|  | R:GGCGTACAGGTCTTTGCGGATG |
| tRF3a-MetCAT | RT:GTCGTATCCAGTGCAGGGTCCGAGGTATTCGCACTGGATACGACTGGTGC |
|  | FP:GAGTGTACCTCAGAGGGG |
|  | RP:AGTGCAGGGTCCGAGGTATT |
| tRF3b-MetCAT | RT:GTCGTATCCAGTGCAGGGTCCGAGCTATTCGCACTGGATACGACTGGTGCC |
|  | FP:GAGTGTCAAGCCTCAGAGGG |
|  | RP:AGTGCAGGGTCCGAGGTATT |
| tRF3-36-MetCAT | RT:GTCGTATCCAGTGCAGGGTCCGAGGTATTCGCACTGGATACGACGTGCCC |
|  | FP:GTCCTGAGTTCGAACCTCAGAGG |
|  | RP:AGTGCAGGGTCCGAGGTATT |
| tRF3-38-MetCAT | RT:GTCGTATCCAGTGCAGGGTCCGAGGTATTCGCACTGGATACGACTGCCCCCT |
|  | FP:TCTGAAGGTCCTGAGTTCGAAC |
|  | RP:AGTGCAGGGTCCGAGGTATT |
|  |  |
| siRNA | |
| STAT1-siRNA-1 | 5'-GCUGGCCCUGAUGGUCUUATT-3' |
|  | 3'-UAAGACCAUCAGGGCCAGCTT-5' |
| STAT1-siRNA-2 | 5'-CCCUGAAGUAUCUGUAUCCAATT-3' |
|  | 3'-UUGGAUACAGAUACUUCAGGGTT-5' |
|  |  |
| shRNA | |
| TRIM21-shRNA-1 |  |
| ccggGAGTTGGCTGAGAAGTTGGAActcgagTTCCAACTTCTCAGCCAACTCtttttg | |
| TRIM21-shRNA-2 |  |
| ccggTGGCATGGTCTCCTTCTACAActcgagTTGTAGAAGGAGACCATGCCAtttttg | |
|  |  |
| ChIP-PCR | |
| Human C5 Promotor | F: GACGCTCACCGCCTAGAAG |
|  | R: CAACCGGCACAACACAACAA |
|  |  |
| tRF3a-MetCAT | Mimic: 5'-ACCUCAGAGGGGGCACCA-3' |
|  | Inhibitor:5'-UGGUGCCCCCUCUGAGGU-3' |
|  | Agomir: 5'-ACCUCAGAGGGGGCACCA-3' |
|  | Antagomir:5'-UGGUGCCCCCUCUGAGGU-3' |
|  |  |
| Negative Control | Mimic:5'-UUUGUACUACACAAAAGUACUG-3' |
|  | Inhibitor:5'-CAGUACUUUUGUGUAGUACAAA-3' |
|  | Agomir:5'-UUUGUACUACACAAAAGUACUG-3' |
|  | Antagomir:5'-CAGUACUUUUGUGUAGUACAAA-3' |

Table S3. Details of antibodies used in this research

| Antibodies List | | |
| --- | --- | --- |
| Antibody | Company | Cat No |
| Anti-C5a antibody | abcam | Cat#ab281923； |
| C5aR Polyclonal antibody | Proteintech | Cat#21316-1-AP; |
| p44/42 MAPK (Erk1/2) (137F5) Rabbit mAb | CST | Cat#4695； |
| Phospho-p44/42 MAPK (Erk1/2) (Thr202/Tyr204) (D13.14.4E) Rabbit mAb | CST | Cat#4370； |
| EGF Receptor (D38B1) XP® Rabbit mAb | CST | Cat#4267； |
| Phospho-EGF Receptor (Tyr1068) (D7A5) XP® Rabbit mAb | CST | Cat#3777； |
| B-Raf (D9T6S) Rabbit mAb | CST | Cat#14814； |
| Phospho-B-Raf (Ser445) Antibody | CST | Cat#2696； |
| MEK1/2 (D1A5) Rabbit mAb | CST | Cat#8727； |
| Phospho-MEK1/2 (Ser217/221) (41G9) Rabbit mAb | CST | Cat#9154； |
| TRIM21 Polyclonal antibody | Proteintech | Cat#12108-1-AP; |
| STAT1 Monoclonal antibody | Proteintech | Cat#66545-1-Ig； |
| HA--probe Antibody (Y-11) | Santa Cruz | Cat#sc-805; |
| HRP-conjugated Affinipure Goat Anti-Rabbit IgG(H+L) | Proteintech | Cat# SA00001-2; |
| HRP-conjugated Affinipure Goat Anti-Mouse IgG(H+L) | Proteintech | Cat# SA00001-1; |
| CoraLite647-conjugated Goat Anti-Mouse IgG (H+L) | Proteintech | Cat# SA00014-10; |
| CoraLite488-conjugated Goat Anti-Rabbit IgG(H+L) | Proteintech | Cat# SA00013-2; |
| HRP-labeled Goat Anti-Rabbit IgG(H+L) | Beyotime | Cat# A0208; |
| DAPI Staining Solution | abcam | Cat# ab228549; |
| PI | Invitrogen | Cat# P1304MP; |
| PE/Cyanine7 Annexin V | BioLegend | Cat# 640949; |
| FITC anti-human CD45 | BioLegend | Cat# 304006; |
| APC anti-human CD68 | BioLegend | Cat# 333809; |
| PE anti-human CD206 (MMR) | BioLegend | Cat# 321105; |
| PE/Cyanine7 anti-human CD80 | BioLegend | Cat# 375408; |

Table S4. Clinical Information of Patients Used for Drug-Resistant Organoid Model Construction (related to Fig1A)

| **NO.** | **Sex** | **Age** | **Pathology** | **EGFR mutation type** | **Stage** |
| --- | --- | --- | --- | --- | --- |
| 1 | Male | 56 | LUAD | Exo21 L858R | IIA |
| 2 | Female | 60 | LUAD | Exo 19del | IB |
| 3 | Female | 64 | LUAD | Exo21 L858R | IB |
